# Mitochondrial protein dysfunction in pathogenesis of neurological diseases

**DOI:** 10.3389/fnmol.2022.974480

**Published:** 2022-09-07

**Authors:** Liang Wang, Ziyun Yang, Xiumei He, Shiming Pu, Cheng Yang, Qiong Wu, Zuping Zhou, Xiaobo Cen, Hongxia Zhao

**Affiliations:** ^1^National Chengdu Center for Safety Evaluation of Drugs, State Key Laboratory of Biotherapy/Collaborative Innovation Center for Biotherapy, West China Hospital of Sichuan University, Chengdu, China; ^2^School of Life Sciences, Guangxi Normal University, Guilin, China; ^3^Guangxi Universities, Key Laboratory of Stem Cell and Biopharmaceutical Technology, Guangxi Normal University, Guilin, China; ^4^Research Center for Biomedical Sciences, Guangxi Normal University, Guilin, China; ^5^Faculty of Biological and Environmental Sciences, University of Helsinki, Helsinki, Finland

**Keywords:** mitochondrial proteins, neurological diseases, pathogenesis, mitochondrial bioenergetics, mitochondrial dynamics, mitophagy, mtDNA maintenance, mitochondrial import machinery

## Abstract

Mitochondria are essential organelles for neuronal function and cell survival. Besides the well-known bioenergetics, additional mitochondrial roles in calcium signaling, lipid biogenesis, regulation of reactive oxygen species, and apoptosis are pivotal in diverse cellular processes. The mitochondrial proteome encompasses about 1,500 proteins encoded by both the nuclear DNA and the maternally inherited mitochondrial DNA. Mutations in the nuclear or mitochondrial genome, or combinations of both, can result in mitochondrial protein deficiencies and mitochondrial malfunction. Therefore, mitochondrial quality control by proteins involved in various surveillance mechanisms is critical for neuronal integrity and viability. Abnormal proteins involved in mitochondrial bioenergetics, dynamics, mitophagy, import machinery, ion channels, and mitochondrial DNA maintenance have been linked to the pathogenesis of a number of neurological diseases. The goal of this review is to give an overview of these pathways and to summarize the interconnections between mitochondrial protein dysfunction and neurological diseases.

## Introduction

The central nervous system (CNS) is a highly specialized organ and consists of distinct cells, such as neurons, astrocytes, microglia, oligodendrocytes, and ependymal cells, all of which work independently but collectively to perform a variety of neuronal functions (Caruso Bavisotto et al., 2019). As one of the most metabolically active organs in the body, the CNS accounts for only ~2% of total body weight, yet consumes at least 20% of the body’s energy (Sokoloff, 1973). It is thus not surprising that the CNS is densely populated with mitochondria (Magistretti and Allaman, 2015). Growing evidence indicates that mitochondria are important for neural development and activities, including neuronal sprouting and plasticity, synaptic transmission and connection, neural oscillations, and cognition (Misgeld and Schwarz, 2017; Nortley and Attwell, 2017; Fang et al., 2019; Filiou and Sandi, 2019). Therefore, mitochondrial dysfunction has been demonstrated as a key player in the development and progression of many neurological disorders.

Mitochondria perform critical roles beyond simply serving as a powerhouse for producing adenosine triphosphate (ATP) *via* oxidative phosphorylation (OXPHOS). They also play vital roles in calcium (Ca^2+^) buffering, biogenesis of lipids and iron-sulfur [Fe-S] clusters, production of reactive oxygen species (ROS), and apoptosis (Osellame et al., 2012; Rangaraju et al., 2019). To fulfill these functions, mitochondria form highly dynamic and motile networks that undergo constant morphology and positioning changes through fusion and fission (El-Hattab et al., 2018). Mitochondria in neurons are extremely dynamic and move quickly along microtubule tracks to facilitate transport to match energy supply and demand, ensure their distribution to the neuronal periphery, allow the exchange of components between mitochondria, and mediate the removal of damaged mitochondria *via* mitophagy (Seager et al., 2020).

**Figure 1:**
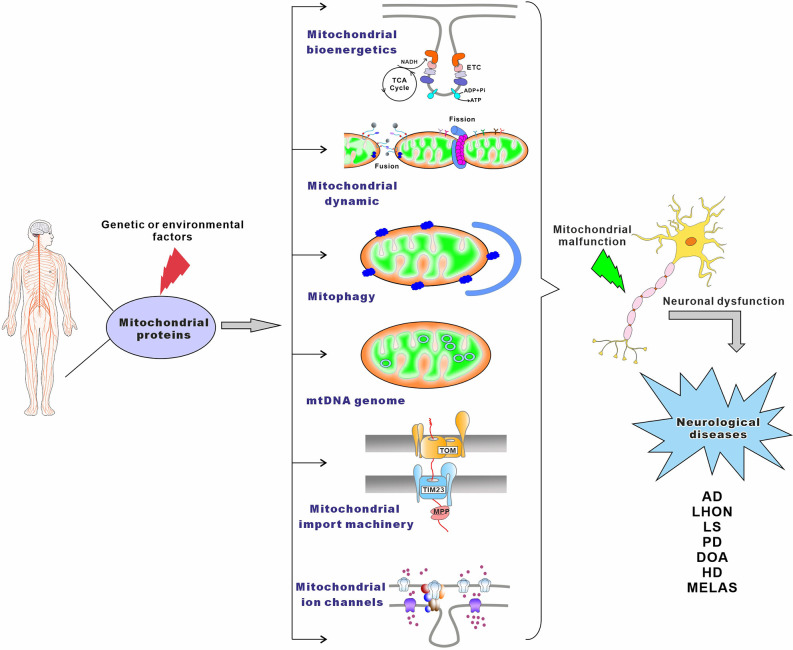
The main pathways leading to mitochondrial-associated neurological disorders. Genetic or environmental factors can cause mutations of mitochondrial proteins, which lead to mitochondrial malfunction. Neurological disorders may result from damaged mitochondrial bioenergetics, imbalanced mitochondrial dynamics, defective mitophagy, impaired maintenance of mitochondrial genome, compromised mitochondrial importing machinery, altered mitochondrial ion channels, or combinations thereof.

A large variety of mitochondrial functions, as well as their morphogenesis and dynamics, have been assigned to mitochondrial proteins and protein complexes (Pfanner et al., 2019). In humans, it is estimated that mitochondria contain at least 1,500 different proteins (Taylor et al., 2003; Lefort et al., 2009). These proteins are involved in mitochondrial bioenergetics, dynamics, mitophagy, import machinery, ion channels, and mitochondrial DNA maintenance (Figure 1). As semi-autonomous organelles, mitochondria encode and synthesize 13 proteins, all of which are core subunits of the respiratory chain. The remaining nearly 99% of mitochondrial proteins are encoded by the nuclear genome, synthesized in the cytosol, and imported into the mitochondria by import, sorting, and assembly machinery (Schmidt et al., 2010). Protein functional analysis shows that approximately 15% of the proteins are directly involved in OXPHOS, 10% of proteins participate in the metabolism of iron, lipids, and amino acids, 20%–25% of proteins maintain and regulate the mitochondrial genome, and about 25% of proteins are involved in a variety of cell signaling, redox, protein and metabolite transport, mitochondrial morphology, and dynamics. Additionally, the cellular roles of almost 20% of mitochondrial proteins still remain unknown (Fox, 2012; Pfanner et al., 2019). The central importance of mitochondrial proteins has been widely highlighted by the association of aberrant mitochondrial protein expression (Lin et al., 2004; Ekstrand et al., 2007; Tysnes and Storstein, 2017), localization (Gandhi, 2006; Lu and Guo, 2020), folding, and function (Castro et al., 2018; Franco-Iborra et al., 2018) with neurological diseases (Figure 1).

Neurological diseases constitute a group of disorders characterized by a progressive deterioration of the brain and spinal cord functions (Farooqui, 2019). Common neurodegenerative diseases include Alzheimer’s disease (AD), Parkinson’s disease (PD), Huntington’s disease (HD), amyotrophic lateral sclerosis (ALS), and multiple sclerosis (Bredesen et al., 2006; Fu et al., 2018). Neuropsychiatric diseases mainly refer to the abnormalities in the cerebral cortex and limbic system, including both neurodevelopmental disorders and behavioral or psychological difficulties associated with some neurological disorders (Farooqui, 2019). Common neuropsychiatric diseases are autism, depression, schizophrenia, and some forms of bipolar disorders, such as depression and anxiety (Lyalina et al., 2013).

In this review, we focus on the association of mitochondrial protein dysfunction with the pathogenesis of neurological diseases, such as AD, PD, and ALS, as well as psychiatric disorders like depression, anxiety, and schizophrenia. Based on the functional classification of mitochondrial proteins, we begin by introducing their key roles in mitochondria. We then incorporate new evidence demonstrating that abnormal mitochondrial protein causes dysregulation of mitochondrial morphology, dynamics, and function in neurological disorders. The literature reviewed here is not exhaustive, but it highlights major findings that characterize the current status of the field in terms of protein-linked mitochondrial quality control in the CNS.

## Dysregulation of Mitochondrial Bioenergetics

The human brain is particularly reliant on a steady supply of energy, almost entirely in the form of glucose, to meet its high metabolic demands (Harris et al., 2012; Nortley and Attwell, 2017). Among all brain cell types, neurons are the principal consumers, using 80%–90% of the total brain energy (Howarth et al., 2012; Zimmer et al., 2017). However, under basal conditions, neurons only uptake almost an equal amount of glucose as astrocytes, and their uptake is significantly lower in the functional activation state (Magistretti and Allaman, 2018). Unlike neurons, which synthesize ATP through OXPHOS, the most effective ATP-producing pathway, astrocytes predominantly metabolize glucose through aerobic glycolysis, resulting in a huge amount of lactate and pyruvate generation from glucose. The produced lactate can be transported to neurons by monocarboxylate transporters (MCTs) and hydrocarboxylic acid receptor 1 (HCAR1), where it serves as an energy substrate for OXPHOS (Morland et al., 2015; Sotelo-Hitschfeld et al., 2015; Díaz-García et al., 2017; Magistretti and Allaman, 2018).

**Figure 2:**
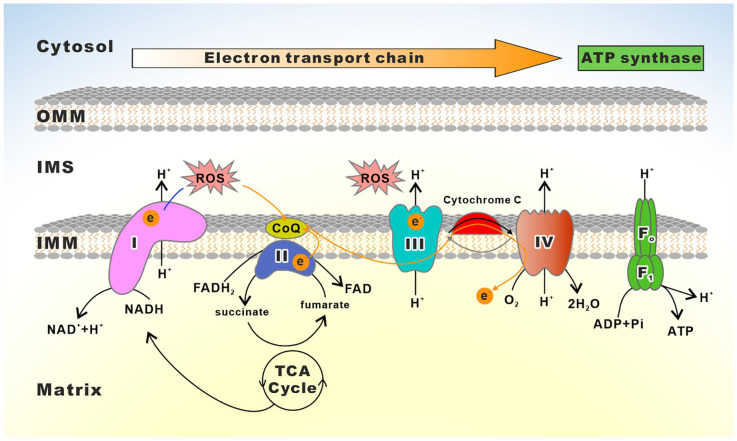
Schematic depiction of the mitochondrial electron transport chain. The electron transport chain (ETC) consists of complexes I to IV as well as two free electron carriers, CoQ and Cyt *c*. Reducing equivalents (NADH, FADH2) provide electrons flowing through complexes I, the ubiquinone cycle (Q/QH2), III, IV, and to the final acceptor O_2_. The electron flows through complexes resulting in the pumping of protons to the IMS, which creates a membrane potential used by the F_1_F_o_-ATP synthase to drive the production of ATP.

OXPHOS occurs *via* the electron transport chain (ETC), which transfers electrons from donors like NADH and FADH2 to the oxygen (MITCHELL, 1961; Cogliati et al., 2021). ETC consists of four enzyme complexes that all reside in the inner mitochondrial membrane (IMM): complexes I (NADH ubiquinone oxidoreductase), II (succinate ubiquinone oxidoreductase), III (ubiquinone cytochrome *c* oxidoreductase or cytochrome *bc*1 complex), and IV (cytochrome c oxidase, COX; Breuer et al., 2013). During electron transfer, three of these complexes (complexes I, III, and IV) pump protons from the matrix to the mitochondrial intermembrane space (IMS), thereby generating an electrochemical proton gradient across the IMM that the F_1_F_o_-ATP Synthase (also known as ATPase Synthase or complex V) exploits to drive ATP synthesis from adenosine diphosphate (ADP; Breuer et al., 2013; Guo R. et al., 2018; Signes and Fernandez-Vizarra, 2018; Nolfi-Donegan et al., 2020). The fundamental role of ETC in mitochondrial energy production is essential for neuronal survival and activity (Li et al., 2021a). However, both complexes I and III can also transfer single electrons to oxygen, resulting in the formation of oxygen superoxide in the mitochondrial matrix. Consequently, mitochondrial DNA (mtDNA), lipids, and proteins including subunits of the OXPHOS complexes exposed to this region experience oxidative damage from the superoxide radical (Dröse and Brandt, 2012; Lenaz, 2012). Thus, dysfunctions in the ETC not only reduce energy production but also result in the excessive accumulation of ROS, which are involved in the development of neurological illnesses, such as PD, AD, ALS, Leigh Syndrome (LS), and HD (Angelova and Abramov, 2018; Pepperberg, 2019; Bakare et al., 2021). Mounting evidence demonstrates that primary mitochondrial disorder-mediated human diseases often arise from mutations in the OXPHOS subunits or the proteins and RNAs required for their expression (Craven et al., 2017; Figure 2 and Table 1).

**Table 1:** Neurological diseases caused by protein variants involved in mitochondrial bioenergetics.

**Protein name**	**Functions**	**Neurological-linked variants**	**Neurological disorders**	**References**
**Complex I**
NDUFS4	Supernumerary subunit	p.Asp60_Lys175del p.Ser34IlefsX5 p.Trp97X p.Arg106X p.Asp119His p.Lys154AsnfsX35	LS Leigh-like syndrome	Quintana et al. (2010, 2012), Assouline et al. (2012), Ortigoza-Escobar et al. (2016), Grillo et al. (2021), and Inak et al. (2021)
NDUFV1	Core subunit	p.ArgR88Gly p.Arg199Pro p.Val245Met p.Thr253Glnfs*44 p.Arg386Cys p.Leu412Pro p.Gly218Cys p.Phe373Leu	LS LBSL Leukoencephalopathy	Marin et al. (2013), Zhang et al. (2019), Borna et al. (2020), and Lee et al. (2020)
NDUFS1	Core subunit	p.Leu231Val p.Thr171Asn p.Asp252Tyr p.Arg518GlyfsX6 p.Thr368Pro P.Arg419Gln p.Tyr209X p.Ala538Pro p.Gln618X p.Val36Phe p.Lys303Ile p.Asp574Val p.Asp574ValfsX10	LS Leukoencephalopathy	Martín et al. (2005), Zhang et al. (2019), and Lee et al. (2020)
NDUFS2	Catalytic core subunit	p.Glu104Ala	LS PD	Marin et al. (2013) and González-Rodríguez et al. (2021)
MT-ND1	Core subunit	m.3460G > A m.3635G>A m.3697G>A	LS LHON	Riordan-Eva and Harding (1995b), Yang et al. (2009), Catarino et al. (2017), and Lee et al. (2020)
MT-ND3	Core subunit	m.10158T>C m.10191T>C m.10197G>A	LS LHON MELAS	Wang et al. (2009), Kori et al. (2019), and Lee et al. (2020)
MT-ND4	Core subunit	m.11778G > A	LHON	Riordan-Eva and Harding (1995b) and Catarino et al. (2017)
MT-ND5	Core subunit	m.13513G>A m.13708G>A	LS LHON	Brown et al. (1992) and Lee et al. (2020)
MT-ND6	Core subunit	m.14459G>A m.14484T > C m.14487T>C	LS LHON	Riordan-Eva and Harding (1995b), Catarino et al. (2017), and Lee et al. (2020)
NDUFA12L	Assembly factor	p.Met1Leu	Ataxia Encephalopathy Tauopathy	Barghuti et al. (2008) and Lazarou et al. (2009)
NDUFAF12	Assembly factor	Del.NDUFAF12 p.Tyr38X	ADHD	Hoefs et al. (2009), Lesch et al. (2011), and Schlehe et al. (2013)
**Complex II**
SDHA	Core subunit	p.Arg451Cys, p.Ala524Val, p.Arg554Trp p.Gly555Glu	LS	Bourgeron et al. (1995), Parfait et al. (2000), Van Coster et al. (2003), and Lake et al. (2016)
SDHB	Core subunit	p.Asp48Val p.Ala102Thr p.Arg230His p.Leu257Val	LS Leukoencephalopathy	Alston et al. (2012), Ardissone et al. (2015), Helman et al. (2016), and Kaur et al. (2020)
SDHD	Core subunit	p.Glu69Lys p.*164Lext*3	Encephalomyopathy	Jackson et al. (2014) and Lin et al. (2021)
SDHAF1	Assembly factor	p.Gly57Arg p.Arg55Pro	LS Leukoencephalopathy	Bourgeron et al. (1995), Ghezzi et al. (2009), and Lake et al. (2016)
**Complex III**
MT-CYB	Catalytic core subunit	m.15257G>A m.15812G>A	LHON	Brown et al. (1992)
UQCRQ	Supernumerary subunit	p.Ser45Phe	Psychomotor retardation Dementia with defects in verbal and expressive communication skills	Barel et al. (2008)
UQCRC2	Supernumerary subunit	p.Gly222Ala	Encephalomyopathy	Burska et al. (2021)
CYC1	Catalytic core subunit	p. Arg317Trp	ADS LHON	Heidari et al. (2021)
BCS1L	Assembly factor	p.Ser78Gly p.Pro99Leu p.Arg155Pro p.Arg184Cys p.Ser277Asn p.Leu280Phe p.Val353Met	Movement disorders Seizures Björnstad syndrome Leigh-like syndrome Encephalopathy	de Lonlay et al. (2001), Baker et al. (2019), and Hikmat et al. (2021)
LYRM7	Assembly factor	p.Asp25Asn p.Lys82Asnfs*10 p.Leu66dup p.Asp25Asn p.Thr13Hisfs*17 Splicing mutation	Encephalopathy Leukoencephalopathy	Invernizzi et al. (2013), Dallabona et al. (2016), Zhang et al. (2019), and Natarajan et al. (2021)
TTC19	Assembly factor	p.Leu219X p.Gln173X p.Gln173ArgfsX4 p.Trp186X p.Gly322MetfsX8 p.Gln77ArgfsX30 p.Ala321fsX8 p.Gln277X p.Pro54AlafsX48 p.Arg194Asnfs*16	Encephalopathy Psychiatric symptoms Cognitive impairment LS Cerebellar ataxia	Ghezzi et al. (2011), Atwal (2013), Nogueira et al. (2013), Melchionda et al. (2014), Kunii et al. (2015), and Habibzadeh et al. (2019)
UQCC2	Assembly factor	p.Arg8Pro p.Leu10Phe	Encephalomyopathy	Feichtinger et al. (2017)
UQCC3	Assembly factor	p.Val20Glu	Delayed psychomotor development	Wanschers et al. (2014)
**Complex IV**
MT-CO3	Core subunit	p.Trp116X	MELAS syndrome	Wang et al. (2021)
NDUFA4	Supernumerary subunit	Splicing mutation	LS LS-like syndrome AD	Coskun et al. (2012) and Pitceathly et al. (2013)
COX4I	Supernumerary subunit	p.Pro152Thr	LS LS-like syndrome Seizures	Pillai et al. (2019)
COX8A	Supernumerary subunit	p.Glu39Argfs*27	LS-like syndrome Epilepsy	Hallmann et al. (2016)
COX5A	Supernumerary subunit	-	AD Memory Impairment Brain aging	Xiyang et al. (2020)
COX6A	Supernumerary subunit	Splicing mutation	CMT	Tamiya et al. (2014)
COX6B	Supernumerary subunit	p.Arg20His p.Arg20Cys	Encephalomyopathy	Massa et al. (2008) and Abdulhag et al. (2015)
COX20	Assembly factor	p.Thr52Pro p.Lys14Arg p.Gly114Ser p.Trp74Cys	Sensory neuropathy Cerebellar ataxia Intellectual disability	Otero et al. (2018)
SURF1	Assembly factor	p.Val177Gly p.Gly257Arg p.Gly199del p.Pro298Leu p.Lys291X p.Pro119Leu p.Glu57Lysfs*16 p.Tyr178Asn p.Pro218Argfs*29 p.Pro218Argfs*30 p.Gly257Arg p.Gly199del p.Pro104Profs*1 p.Ser282Cysfs*9	LS Neurodegeneration Optic atrophy Seizure Ataxia	Danis et al. (2018), Kose et al. (2020), Inak et al. (2021), and Lee and Chiang (2021)
COA3	Assembly factor	p.Leu67Profs*21 p.Tyr72Cys	Neuropathy	Ostergaard et al. (2015)
COA7	Assembly factor	p.Asp6Gly p.Arg39Trp p.Tyr137Cys p.Gly144fs p.Ser149Ile p.Ala171Thr p.Trp185* p.Asn189Ser Splicing mutation	Cerebellar ataxia Cognitive impairment Cerebellar atrophy Leukoencephalopathy SCAN3	Martinez Lyons et al. (2016), Higuchi et al. (2018), and Ban et al. (2021)
SCO2	Assembly factor	p.Asp135Gly p.Glu140Lys p.Pro169Thr p.Arg171Gln	CMT	Rebelo et al. (2018)
LRPPRC	Assembly factor	p.Tyr172Cys	LS	Kotecha and Kairamkonda (2019)
COX10	Assembly factor	p.Asn204Lys p.Thr196Lys p.Pro225Leu p.Asp336Val/Gly	Encephalopathy LS	Valnot (2000) and Antonicka (2003)
COX15	Assembly factor	p.Arg217Trp p.Leu139Val	LS	Oquendo (2004) and Miryounesi et al. (2016)
TACO1	Assembly factor	p.Glu226Ter p.Tyr278Cys p.His158ProfsX8 p.Cys85PhefsX15 p.Ile164Asn	Leukoencephalopathy Optic atrophy Visual impairment	Oktay et al. (2020) and Sferruzza et al. (2021)
PET100	Assembly factor	p.Met1?	LS	Lim et al. (2014)
FASTKD2	Assembly factor	p.Leu270 fs p.Arg290X p.Ser621Leufs*	Dyscinesia Optic atrophy Stroke	Wei et al. (2020a)
PET117	Assembly factor	p.Gln58X	Neurodevelopmental regression	Renkema et al. (2017)
**F1Fo-ATP Synthase**
MT-ATP6	Core subunit	p.Pro12Arg p.Gly16Ser p.Pro18Ser p.Pro66Ala p.Thr96Ala p.Ala155Pro p.Leu156Arg p.Gly167Asp p.Leu170Pro p.Gln210X p.Leu217Pro p.Leu220Pro	LS NARP LHON Axonal neuropathy Peripheral neuropathy Cerebellar atrophy Spinocerebellar ataxia syndromes	Jonckheere et al. (2012), Pfeffer et al. (2012), Duno et al. (2013), Claeys et al. (2016), Kytövuori et al. (2016), Nolte et al. (2021), and Kenvin et al. (2022)
MT-ATP8	Supernumerary subunit	p.Pro10Ser p.Pro12Arg p.Pro66Ala	LS Encephalopathy Peripheral neuropathy	Kytövuori et al. (2016) and De Michele et al. (2018)
ATP5E	Core subunit	p.Tyr12Cys	Mental retardation Peripheral neuropathy	Mayr et al. (2010)
ATP5A1	Core subunit	p.Arg329Cys	Encephalopathy	Jonckheere et al. (2013)
ATPAF2 (ATP12)	Assembly factor	p.Trp94Arg	Encephalomyopathy	De Meirleir (2004) and Kanungo et al. (2018)
TMEM70	Assembly factor	p.Tyr179His Splicing mutation	Congenital cataract Neonatal mitochondrial encephalo-cardiomyopathy	Čížková et al. (2008) and Atay et al. (2013)

### Complex I

Complex I (CI) is the first enzyme of ETC, pumping four protons into the IMS upon each NADH oxidation in the matrix and transferring two electrons to ubiquinone in the IMM *via* flavin mononucleotide and a series of iron-sulfur centers (Walker, 1992). Being the largest component, CI is comprised of more than 45 subunits, with seven subunits (MT-ND1- MT-ND6 and MT-ND4L) encoded by mtDNA (Sazanov, 2015). Fourteen CI subunits, which are highly conserved from prokaryotes to humans, constitute the minimal working core of the enzyme and harbor all the redox and proton-motive activity. These supernumerary subunits are thought to increase genetic complexity, susceptibility, and phenotypic diversity to some extent (Carroll et al., 2006; Papa et al., 2012; Sánchez-Caballero et al., 2016). As the point of entry in the mitochondrial ETC for NADH reducing equivalents, CI acts as a regulatable pacemaker for respiratory ATP synthesis in mammalian cells. Given the importance of CI in energy provision, any mutation that disrupts its structure or function seriously perturbs the ETC, resulting in a reduction of ATP synthesis and an accumulation of ROS and other reactive species (Fiedorczuk and Sazanov, 2018; Yin et al., 2021). Lots of studies have discovered that a large proportion of mitochondrial-related diseases are caused by mutations or chemical inhibition of the mitochondrial CI (Papa and De Rasmo, 2013; Rodenburg, 2016; Abramov and Angelova, 2019; Holper et al., 2019; González-Rodríguez et al., 2021).

NDUFS4, an accessory subunit of CI, plays an essential role in the assembly and stability of the CI (Scacco et al., 2003; Kahlhöfer et al., 2017). At the clinical level, mutations in NDUFS4 severely affect the brainstem and basal ganglia, which are typically associated with hypotonia, abnormal ocular movements, visual impairment, psychomotor arrest/regression, and episodes of respiratory failure. Children with NDUFS4 mutations (such as p.97Ter) show a homogeneous early-onset LS and a severe, lethal course of the disease (Ortigoza-Escobar et al., 2016). Additionally, the NDUFS4 knockout mouse (*ndufs4*^−/−^) is one of the most widely used mouse models for investigating the pathomechanism of LS, a rare, complicated, and incurable early-onset pediatric disorder with both phenotypic and genetic heterogeneity featuring brain-specific anomalies (Quintana et al., 2010, 2012; Grillo et al., 2021). Except for NDUFS4, the mutations in other components of CI, such as NDUFV1 and NDUFS1, have also been linked to LS (Martín et al., 2005; Marin et al., 2013; Borna et al., 2020). One recent study on 37 children with cavitating leukoencephalopathies (a brain disorder that predominantly damages the cerebral white matter) was found caused by mutations in NDUFV1 (2/37) and NDUFS1 (5/37; Zhang et al., 2019). Besides LS, one infant with a biallelic loss-of-function mutation in NDUFV1 is also compatible with the phenotype of LBSL (Leukoencephalopathy with brainstem and spinal cord involvement and lactate elevation), a rare condition characterized by progressive pyramidal, cerebellar, and dorsal column dysfunction (Borna et al., 2020). Moreover, partially disrupted CI function in dopaminergic neurons by deleting its NDUFS2, an essential subunit for the catalytic core of CI, caused progressive, human-like parkinsonism in which the loss of nigral dopamine release makes a critical contribution to motor dysfunction (González-Rodríguez et al., 2021).

Besides the nuclear DNA (nDNA) encoded subunits, aberrant mtDNA-encoded components, MT-ND1, MT-ND3, MT-ND4, MT-ND5, and MT-ND6, caused the dysfunction of CI that is also involved in the pathomechanism of neurological disorders, such as LS and Leber hereditary optic neuropathy (LHON; Catarino et al., 2017; Lee et al., 2020; Bakare et al., 2021). For instance, the MT-ND3 mutation disrupted the active/inactive transition of CI, and the MT-ND5 mutation inhibited proton translocation, both of which were found in patients with LS and LHON (Wang et al., 2009; Vodopivec et al., 2016; Lee et al., 2020). Additionally, an m.10158T>C mutation in MT-ND3 has also been linked to mitochondrial encephalomyopathy with lactic acid and stroke-like episodes (MELAS) syndrome (Kori et al., 2019). Three primary point mutations in mtDNA, m.3460G>A, m.11778G>A, and m.14484T>C, have been shown to be responsible for more than 95% of LHON patients (Riordan-Eva and Harding, 1995a; Catarino et al., 2017).

Observations from the mammalian CI suggest an “L” shaped structure with a hydrophilic arm protruding into the matrix and a hydrophobic arm embedding within the IMM (Wirth et al., 2016; Zhu et al., 2016; Parey et al., 2020). This highly complicated structure with over 45 subunits requires at least 15 assembly factors for its complete maturation (Andrews et al., 2013; Formosa et al., 2018; Parey et al., 2020). Hence, mutations in the genes encoding assembly factors lead to a reduced amount of fully assembled functional CI by affecting the rate of CI stability (Bakare et al., 2021). For example, Barghuti et al. (2008) discovered that one CI assembly factor NDUFA12L is mutated in two patients who presented in late infancy with hypotonia, nystagmus, and ataxia interspersed with acute episodes of encephalopathy (Barghuti et al., 2008). Additionally, mutations in NDUFA12L may also have a functional impact on the etiology of tauopathies, suggesting that NDUFA12L could be a crucial factor in the development of neurodegenerative tauopathy, a possible contributing factor in the development of PD (Salama and Mohamed, 2015). In addition, NDUFS4 deletion also results in the nearly complete absence of another accessory subunit NDUFAF12 (Adjobo-Hermans et al., 2020). In NDUFAF12-mutated patient cells, the level of assembled and active CI is reduced, eventually leading to mitochondrial deterioration by increasing mitochondrial ROS and damaging mtDNA and lipids (Schlehe et al., 2013). A heterozygous NDUFAF12 deletion has been observed in a patient with attention-deficit/hyperactivity disorder (ADHD), a common, highly heritable neurodevelopmental syndrome characterized by hyperactivity, inattention, and increased impulsivity (Lesch et al., 2011).

### Complex II

Complex II (CII), also known as succinate dehydrogenase (SDH), is the smallest complex of the ETC. This complex is composed of only four subunits SDHA, SDHB, SDHC, and SDHD, which are all encoded by the nuclear genome (Sun et al., 2005; Bezawork-Geleta et al., 2017). Moreover, four assembly factors are required for the assembly and maturation of CII (Bezawork-Geleta et al., 2017). As part of ETC, CII transfers electrons from succinate to ubiquinone (UbQ) *via* its [Fe-S] clusters. SDHA, the largest catalytic subunit of CII, oxidizes succinate and couples this to the reduction of its flavine cofactor, FAD; whereas the other catalytic subunit SDHB shuttles electrons to UbQ in a coordinated manner (Cecchini, 2003; Miyadera et al., 2003). In addition to its role in ETC, CII also participates in the tricarboxylic acid cycle (TCA), forming a functional link between these two vital processes (Cecchini, 2003; Cardaci et al., 2015; Lussey-Lepoutre et al., 2015).

Although fewer neurological disorders are reported to be linked to the mutations in CII, multiple types of mutations in SDHA, and SDHB, as well as one assembly factor SDHAF1, have been reported to cause LS or LS-like symptoms (Bourgeron et al., 1995; Birch-Machin et al., 2000; Pagnamenta et al., 2006; Jain-Ghai et al., 2013; Bezawork-Geleta et al., 2017; Kaur et al., 2020). Mutations in the *SDHA* gene are the most commonly reported cause of isolated complex II deficiency (Fullerton et al., 2020). People who carry one of the SDHA mutations, p.R451C, p.A524V, p.R554W, or p.G555E mutations, are diagnosed with LS (Bourgeron et al., 1995; Parfait et al., 2000; Van Coster et al., 2003). Except for LS, multiple point mutations in SDHB are reported in patients with leukoencephalopathy (Alston et al., 2012; Ardissone et al., 2015; Helman et al., 2016; Kaur et al., 2020). In patients with early progressive encephalomyopathy, a homozygous or compound heterozygous mutations in SDHD p.E69K and p.*164Lext*3 (a mutation that extends the SDHD C-terminus by the three extra amino acids Leu, Pro, and Phe) was identified (Jackson et al., 2014; Lin et al., 2021). Moreover, although CI and CIII are the main sources of mitochondrial ROS, growing studies indeed reveal that CII also serves as a source and modulator of ROS (Yankovskaya et al., 2003; Hadrava Vanova et al., 2020). Both functional loss of CII and its pharmacological inhibition lead to excessive ROS accumulation, which has a relevant impact on the development of pathophysiological conditions such as neurodegenerative disorders (Lin and Beal, 2006; Iverson et al., 2012).

### Complex III

Complex III (CIII) catalyzes the transfer of electrons from ubiquinol (CoQH2) to cytochrome *c* (Cyt *c*), as well as couples this electron transfer to the vectorial translocation of two protons from the matrix, releasing four into the IMS (Zhang et al., 1998; Meunier et al., 2013). CIII forms a homodimeric complex consisting of 10–11 subunits per monomer, and the biogenesis of CIII requires approximately eight assembly factors (Ndi et al., 2018; Vercellino and Sazanov, 2022). Except for MT-CYB encoded by mtDNA, the remaining subunits are encoded by nDNA. The enzyme has two distinct quinone-binding sites (Q_o_ or Q_P_, quinol oxidation site, and Q_i_ or Q_N_, quinone reduction site), which are located on opposite sides of the IMM and linked by a transmembrane electron-transfer pathway. Each monomer of the CIII contains three catalytic subunits that are highly conserved from prokaryotes to eukaryotes: MT-CYB, cytochrome c1 (also known as CYC1), and the Rieske iron-sulfur protein (UQCRFS1; Xia et al., 2013). MT-CYB provides both the Q_o_ and Q_i_ pockets and the transmembrane electron pathway (*via* hemes b_L_ and b_H_; Xia et al., 1997; Iwata et al., 1998).

Mutations in subunits of CIII (MT-CYB, UQCRB, UQCRQ, UQCRC2, CYC1, and UQCRFS1) and its assembly factors (BCS1L, LYRM7, TTC19, UQCC2, UQCC3) lead to inherited disorders in humans (Fernández-Vizarra and Zeviani, 2015; Feichtinger et al., 2017). A homozygous missense mutation of CYC1 (p.R317Y) caused CIII deficiency, resulting in mitochondrial respiratory chain abnormalities and eventually leading to acute demyelinating syndrome or LHON (Heidari et al., 2021). Another homozygous missense (p.S45F) mutation in UQCRQ, one of the CIII subunits that joins and stabilizes the mature MT-CYB by forming a stable intermediate complex together with UQCRB (Gruschke et al., 2012; Vercellino and Sazanov, 2022), caused the reduced activity of CIII and CI. This defect in OXPHOS, especially for CIII, causes severe neurological disorders characterized by severe psychomotor retardation and global dementia with defects in verbal and expressive communication skills (Barel et al., 2008). Another homozygous missense mutation (p.G222A) in one core subunit UQCRC2, which plays an important role in CIII dimerization during the early period of the CIII assembly (Stephan and Ott, 2020), also causes neurological disorders, presenting with severe encephalomyopathy, paleocerebellar symptomatology, delay in motoric and cognitive functions (Burska et al., 2021). Additionally, one girl with two homozygous missense variations in UQCC2 leads to a severe reduction of UQCC2 protein, a key assembly factor that regulates MT-CYB expression and subsequent complex III assembly (Tucker et al., 2013). The reduced UQCC2 inhibits the activity of CI and CIII, leading to the progression of epileptic seizures (Feichtinger et al., 2017). Furthermore, patients with homozygous mutations in another assembly factor TTC19 (tetratricopeptide repeat domain 19), such as p.L219*, p.E173*, are found to develop a series of neurodegenerative disorders, including severe psychiatric symptoms, cerebellar ataxia, cognitive impairment, LS and so on (Ghezzi et al., 2011; Atwal, 2013; Malek et al., 2018; Habibzadeh et al., 2019).

### Complex IV

Complex IV (CIV) is the terminal enzyme of the ETC, catalyzing the transfer of electrons from reduced Cyt *c* to molecular oxygen. It is comprised of 14 subunits, three (MT-COX1, MT-COX2, and MT-COX3) of which are encoded by the mtDNA. These three mtDNA-encoded subunits form the catalytic fraction of CIV, and the remaining ten nDNA-encoded subunits contribute to the assembly and biogenesis of the CIV (Antonicka, 2003; Böhm et al., 2006). Although the number of unique subunits between CIII and CIV is similar, CIV requires far more assembly factors for biogenesis than CIII, with almost 50 assembly factors necessary for CIV assembly (Zong et al., 2018; Brzezinski et al., 2021; Vercellino and Sazanov, 2022).

CIV deficiency is the second most common ETC complex deficiency, occurring in 19%–27% of all mitochondrial disease patients (Renkema et al., 2017). The majority of recorded occurrences present a severe, often fatal, infantile disease (Balsa et al., 2012; Pitceathly et al., 2013). Multiple homozygous mutations of CIV subunits, such as NDUFA4 (c.42 + 1G>C, aberrant splicing), COX4I (c.454C>A, p.P152T), and COX8A (c.115-1G>C, p.E39Rfs*27), have been discovered in patients with LS or LS-like syndrome. These mutations cause a frameshift with premature stop-codon formation, which results in truncated proteins or even loss of proteins, leading to the reduced quantity or activity of CIV (Pitceathly et al., 2013; Hallmann et al., 2016; Pillai et al., 2019; Čunátová et al., 2020). Mutation in mtDNA encoded core subunit MT-CO3 (m.9553G>A, p.W116X) was founded in a female patient with the MELAS syndrome (Wang et al., 2021). Except for spontaneous mutations in its subunits, some dysregulated proteins, like β-amyloid (Aβ) peptides, can specifically inhibit the activity of CIV by binding to the heme of CIV and forming an Aβ-heme complex, resulting in cognitive deficits and neuronal morphological abnormalities, which are implicated in the development of AD and PD (Atamna and Boyle, 2006; Coskun et al., 2012). Furthermore, the profound changes in two CIV subunits, COX5A and NDUFA4, also suggest a link between CIV abnormalities and mitochondrial dysfunction in the etiology of AD (Shim et al., 2017). COX6A mutations at various loci have been associated with various forms of neuropathy. For example, mutations in COX6B1 (p.R20H/C) and a 5 bp deletion (c.247-10_247-6delCACTC, aberrant splicing) in a splicing element of COX6A1 cause severe infantile encephalomyopathy and an axonal form of Charcot-Marie Tooth (CMT) syndrome, respectively (Massa et al., 2008; Tamiya et al., 2014; Abdulhag et al., 2015).

The current knowledge of neurological disorders linked to CIV deficits reveals that mutations in assembly factors are far more common than mutations in the structural subunits of the CIV (Renkema et al., 2017). Bi-allelic loss of function variants in the *COX20* (c.41A>G or c.157+3G>C) gene perturbs the assembly of complex IV, which leads to mitochondrial bioenergetic failure, resulting in ataxia and autosomal recessive sensory neuronopathies (Otero et al., 2018; Dong et al., 2021). Except for COX20, defects or mutations in other CIV assembly proteins such as SURF1, COA3, COA7, and SCO2 may impair axonal transport or mitochondrial copper homeostasis, as well as increase ROS damage, resulting in peripheral neuropathies (Echaniz-Laguna et al., 2013; Ostergaard et al., 2015; Higuchi et al., 2018; Rebelo et al., 2018; Finsterer and Winklehner, 2021). Mutations in these assembly factors, which are involved in the biogenesis of CIV, LRPPRC (p.Y172C), COX10, SURF1 (834G→A or 820-824dupTACAT), COX15, TACO1, and PET100 (c.3G>C), have been identified in patients with LS (Bugiani, 2005; Tay et al., 2005; Coenen et al., 2006; Weraarpachai et al., 2009; Lim et al., 2014; Kotecha and Kairamkonda, 2019). Furthermore, a mutation in the *FASTKD2* (fas activated serine-threonine kinase domain 2) gene induces the occurrence of epilepsy in infancy (Ghezzi et al., 2008). Patients with bradykinesia and cognitive impairment have a homozygous mutation in PET117, a potential complex IV assembly component (Renkema et al., 2017; Finsterer and Winklehner, 2021).

### F_1_F_o_-ATP Synthase

F_1_F_o_-ATP synthase is the terminal complex of the OXPHOS and thus is also referred to complex V (CV). F_1_F_o_-ATP synthase is composed of 19 structural subunits, two of which (MT-ATP6 and MT-ATP8) are encoded by mtDNA and the other 17 by nDNA. Five assembly factors are involved in the biogenesis of this multi-subunit enzyme (Genov et al., 2020; Pinke et al., 2020; Vercellino and Sazanov, 2022). Based on the structural and functional differences, F_1_F_o_-ATP synthase is divided into two domains: a hydrophobic domain embedded in the IMM (Fo) is responsible for proton translocation by forming a rotatory proton channel, and a hydrophilic ATPase domain (F1) exposed to the matrix synthases ATP by phosphorylating ADP to ATP (MITCHELL, 1961; Zhou and Sazanov, 2019; Spikes et al., 2020). Mutations in either of the nuclear or mitochondrial encoded subunits may damage the activity of F_1_F_o_-ATP Synthase, resulting in reduced energy production (Kucharczyk et al., 2009; Ebanks et al., 2020; Mnatsakanyan and Jonas, 2020; Patro et al., 2021).

ATP synthase deficiency-caused illnesses are often severe encephalopathies or cardiomyopathies and appear soon after birth (Dautant et al., 2018). The better-characterized aberrant ATP synthase-caused diseases are attributed to mutations in the mtDNA-encoded *MT-ATP6* and *MT-ATP8* genes, which have overlapped 46 nucleotides at the 5’ part of *MT-ATP6* with the open reading frame of *MT-ATP8* (Taanman, 1999). As a result, alterations in this region probably cause double mutations (Ware et al., 2009; Imai et al., 2016; Fragaki et al., 2019; Galber et al., 2021). For instance, the heteroplasmic m.8561C>G or m.8561C>T mutations in the overlapping region of MT-ATP6 and MT-ATP8 in adult and childhood caused severe neurological signs, presenting with cerebellar ataxia, psychomotor delay, peripheral neuropathy, and microcephaly (Kytövuori et al., 2016; Fragaki et al., 2019). Compared to MT-ATP8, mutations in MT-ATP6-caused defects of ATP synthase occur more frequently in humans. Mutations in MT-ATP6, like the most common mutations (m.8993T>G/C and m.9176T>G/C), as well as less frequent mutations (m.9035T>C, m.9185T>C, m.9191T>C, m.8914C>T, m.8701A>G), cause different clinical phenotypes of neuropathy, varying from NARP (Neuropathy, Ataxia, Retinitis Pigmentosa) to, encephalomyopathy and MILS (Maternally Inherited Leigh’s Syndrome; Guo Y. et al., 2018; Ichikawa et al., 2019; Capiau et al., 2022; Na and Lee, 2022). Besides mutations in the mtDNA encoded subunits, ATP synthase deficits induced by nDNA-encoded subunits, such as ATP5E and ATP5A1, as well as the assembly factors ATPAF2 (ATP12) and TMEM70, have also been observed in patients with neurological disorders (De Meirleir, 2004; Čížková et al., 2008; Mayr et al., 2010; Jonckheere et al., 2013). For example, exome sequencing of the two siblings with severe neonatal encephalopathy reveals a heterozygous mutation in CV subunit ATP5A1, resulting in a disrupted inter-subunit interaction and thereby compromising CV stability (Jonckheere et al., 2013).

## Dysregulation of Mitochondrial Dynamics

Mitochondria are dynamic organelles that divide and fuse rapidly, which determine the mitochondrial architecture and bioenergetics (El-Hattab et al., 2018). Both the fission and fusion processes are predominately performed by several canonical dynamin-like guanosine triphosphatases (GTPase) enzymes (Trevisan et al., 2018; Sinha and Manoj, 2019). Mitochondrial fusion is primarily accomplished by two outer mitochondrial membrane (OMM) proteins, mitofusin 1 (Mfn1) and mitofusin 2 (Mfn2), and one IMM protein, optic dominant atrophy (OPA1). Mitochondrial fission is mediated by dynamin-related GTPase protein 1 (Drp1) and dynamin 2 (Dnm2), which are responsible for membrane constriction and scission, respectively. In addition, the trans-localization of Drp1 relies on multiple OMM-localized transmembrane receptors like MiD51, MiD49, Fis1, and Mff (Giacomello et al., 2020; Yang et al., 2022). Furthermore, mitochondria actively move along microtubes in both anterograde and retrograde directions with the help of ATP-dependent “motor” proteins (Pozo Devoto and Falzone, 2017). Mitochondrial anterograde transport from the soma to synapses is mediated by kinesins, while retrograde transport from the synapse to the soma is carried out by dynein motor proteins. Mitochondria do not bind directly to the motor proteins but, instead, bind to adaptor proteins, TRAK and Miro, that link the mitochondrial membrane to the motor protein (Maday et al., 2014; Zheng et al., 2019).

**Figure 3:**
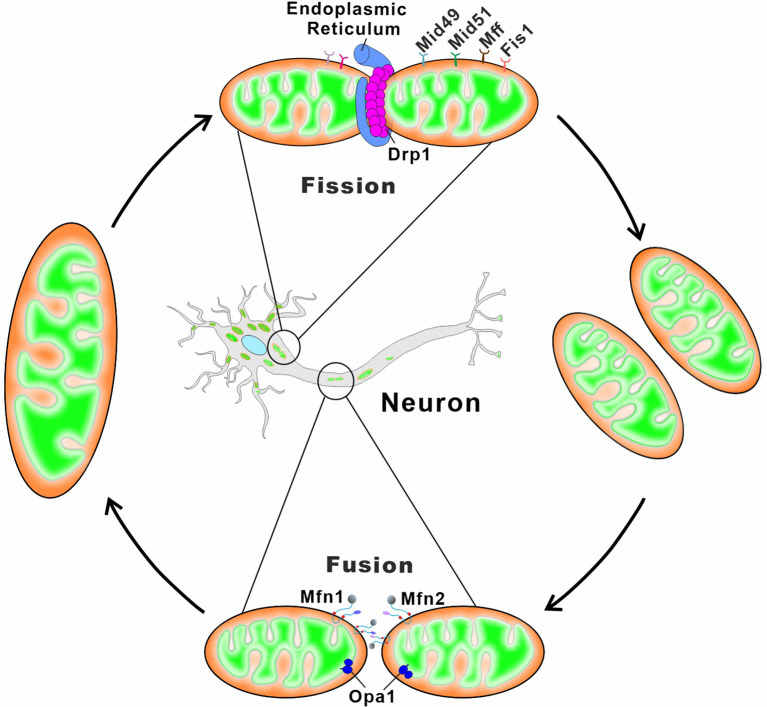
Schematic depiction of mitochondrial fusion and fission in neurons. Mitochondrial fusion relies on membrane proteins Mfn1/2 and OPA1 residing in the OMM and the IMM, respectively. Mfn1 and Mfn2 form homo-oligomeric (Mfn1-Mfn1 or Mfn2-Mfn2) and hetero-oligomeric (Mfn1-Mfn2) complexes in trans between the opposing mitochondria to induce the OMM fusion. The long and short forms of OPA1 synergistically catalyze the tethering and fusion of the IMM. Mitochondrial fission begins with the ER contacts with the OMM at the ER-mitochondria contact sites, and ER wraps tightly around the mitochondria to form constrictions. The cytosolic Drp1 is subsequently recruited to the OMM *via* multiple membrane adaptors MiD51, MiD49, Fis1, and Mff. Drp1 oligomerizes at the ER-marked pre-constriction sites, forming a ring-like structure around the mitochondria for further membrane constriction.

Although mitochondrial biogenesis can occur in the axon, the majority of new mitochondria are produced in the soma and transported to the peripheral area of the distal synapse (Amiri and Hollenbeck, 2008; Sheng and Cai, 2012) As a result, mitochondrial anterograde transport can deliver young mitochondria to distal regions (Farías et al., 2017; Zheng et al., 2019). In addition, aged or damaged mitochondria in the distal region are transported back to soma for complete degradation *via* mitophagy (Zheng et al., 2019; López-Doménech et al., 2021). Neurons are particularly vulnerable to both autophagic impairment and mitochondrial malfunction. Defects in mitochondrial fusion, division, or transportation result in the accumulation of dysfunctional mitochondria, which promotes neurological disease pathogenesis (Martinez-Vicente, 2017; Han et al., 2020; Doxaki and Palikaras, 2021; Figure 3 and Table 2).

**Table 2:** Neurological diseases caused by protein variants involved in mitochondrial dynamics.

**Protein name**	**Pathogenic mutations or changes**	**Neurological disorders**	**References**
**Mitochondrial fusion**
Mfn2	p.Trp740Ser, p.Leu76Pro, p.Arg280His, p.Pro251Ala, p.Arg94Gln, p.Val69Phe, p.Arg94Gln, p.Arg707Trp, p.Gly176Ser p.Arg 468His	CMT disease	Züchner et al. (2004), Finsterer et al. (2018), Iapadre et al. (2018), and Ababneh et al. (2022)
	p.Arg104Trp	Early-onset choreic movement disorder	Pellino et al. (2021)
	p.Asp210Tyr	Optic atrophy “plus” phenotype	Renaldo et al. (2012)
OPA1	p.Val903Glyfs*3, p.Arg290Gln p.Arg857*, p.Asp438Val, p.Arg52* p.Lys212Argfs*4, p.Thr381_Asn404del p.Phe747Leufs*53, p.Arg366* p. Arg247His, p.Gln31*, p.Asp438Gly, p.Arg932Cys	DOA	Amati-Bonneau et al. (2009), Shamseldin et al. (2012), Ścieżyńska et al. (2017), and Mei et al. (2019)
	p.Arg445His, p.Gly401Asp, p.Leu243*, p.Arg437Glu, p.Ala357Leufs*4	Optic atrophy and hearing loss	Zhang et al. (2015)
	p.Arg445His p.Gly401Asp	ADOA and deafness	Amati-Bonneau et al. (2009)
	p.Ser646Leu	ADOA with MS-like features	Amati-Bonneau et al. (2009)
	p.Leu730Ser, p.Val903GlyfsX3 p.Ile382Met, p.Cys551Tyr	Behr syndrome	Bonneau et al. (2015), Carelli et al. (2015b), and Zeng et al. (2020)
	p.Gly488Arg p.Ala495Val	CPEO, PD, and dementia	Carelli et al. (2015a)
SLC25A46	p.Thr142Ile, p.Arg347Cys	LS	Janer et al. (2016) and Li et al. (2021b)
	p.Arg257Gln, p.Gly249Asp p.Arg340Cys, p.Glu335Asp p.Pro333Leu, p.Ser32Thrfs*4 p.Thr142Ile, p.Leu138Arg	Optic atrophy, axonal neuropathy, ataxia, myoclonic jerks	Abrams et al. (2018)
**Mitochondrial fission**
Drp1	p.Ala395Asp	Truncal hypotonia with little spontaneous movement and no tendon reflexes, poor visual fixation	Waterham et al. (2007)
	p.Ser36Gly p.Trp88Metfs* p.Glu116Lysfs*6 p.Glu129Lys*6 p.Thr115Met p.Leu406Ser	Lethal infantile encephalopathy	Nasca et al. (2016), Yoon et al. (2016), Zaha et al. (2016), and Hogarth et al. (2018)
	p.Gly32Ala	Developmental delay, sensory neuropathy, and optic atrophy	Whitley et al. (2018)
	p.Asp146Asn	Psychomotor developmental delay, and severe ataxia	Longo et al. (2020)
	p.Glu2Ala p.Ala192Glu	DOA	Gerber et al. (2017)
	p.R403C	Epileptic encephalopathy	Fahrner et al. (2016)
Mff	p.Arg145 ×, p.Ser7Phe	Encephalopathy	Agarwal et al. (2020) and Panda et al. (2020)
	p.(Leu62Profs*13; (Arg298*) p.(Glu153Alafs*5);(Glu153Alafs*5) p.(Glu153Alafs*5);(Glu153Alafs*5) p.(Gln64*);(Gln64*)	Seizures, developmental delay and acquired microcephaly, dysphagia, spasticity, and optic and peripheral neuropathy	Shamseldin et al. (2012) and Koch et al. (2016)
SLC25A46	p.Thr142Ile, p.Arg347Cys	LS	Janer et al. (2016) and Li et al. (2021b)
	p.Arg257Gln, p.Gly249Asp p.Arg340Cys, p.Glu335Asp p.Pro333Leu, p.Ser32Thrfs*4 p.Thr142Ile, p.Leu138Arg	Optic atrophy, axonal neuropathy, ataxia, myoclonic jerks	Abrams et al. (2018)

### Mitochondrial fusion

#### Mfn1 and Mfn2

The integral mitochondrial outer membrane protein mitofusins Mfn1 and Mfn2 can form both homo-oligomeric (Mfn1-Mfn1 or Mfn2-Mfn2) and hetero-oligomeric (Mfn1-Mfn2) complexes in trans between the opposing mitochondria to induce the OMM fusion (Chen et al., 2003; Griffin and Chan, 2006; Qa et al., 2016). The primary protein sequence, as well as the structure of Mfn1/2, are quite similar, with an N-terminal GTPase domain, two transmembrane domains spanning the OMM, and two regions essential for protein-protein interactions (Santel and Fuller, 2001; Legros et al., 2002; de Brito and Scorrano, 2008; Yang et al., 2022). Despite both proteins being essential for mitochondrial fusion, Mfn1 has considerably higher GTPase activity than Mfn2 (Ishihara et al., 2004). In addition to modulating mitochondrial morphology, Mfn2 also participates in the bridging of mitochondria to the endoplasmic reticulum (ER) as well as multiple signaling pathways, regulating mitochondrial metabolism, apoptosis, and even the cell cycle (de Brito and Scorrano, 2008; Muñoz et al., 2013).

Although the loss of either Mfn1 or Mfn2 causes lethality in mice due to overtly fragmented mitochondria, mutations in human Mfn2 but not Mfn1 lead to Charcot-Marie-Tooth disease type 2A (CMT2A), peripheral neuropathy of long motor and sensory neurons (Züchner et al., 2004). Although no Mfn1 mutation is found in CMT2A patients, Mfn1 but not Mfn2 functionally complements the CMT2A mutant Mfn2^R94Q^ to induce mitochondrial fusion (Detmer and Chan, 2007). Mfn2 ablation mediated aberrant mitochondrial dynamics causes abnormal mitochondrial distribution, ultrastructure, and ETC activity in Purkinje cells, which impairs dendritic outgrowth and spine formation, resulting in cell death and animal movement defects (Chen et al., 2007). Another* in vivo* study found that loss of Mfn2 results in severe, age-dependent motor deficits characterized by reduced activity and rearing, which are preceded by the loss of dopaminergic terminals in the striatum. These nigrostriatal abnormalities may be a risk factor for neurodegeneration in PD (Pham et al., 2012). In addition, the expression of the Mfn2 mutant in the juvenile stage of a mouse results in fatality. However, Mfn2 mutant expression in adulthood does not cause abnormalities until 150 days. The progressive neurodegeneration, irregular behaviors, and learning and memory deficiencies start to emerge after this silent 150-day period, which is similar to those seen in human neurodegenerative diseases (Ishikawa et al., 2019). These findings indicate that defects in Mfn2 cause irregular neuronal mitochondrial dynamics, which not only seriously affect survival during early life stages but also significantly damage brain function and even maturation.

#### OPA1 and OMA1

In 2000, OPA1 mutations, primarily frameshift and missense variants, were first identified as the cause of dominant optic atrophy (DOA), a blinding disease characterized by retinal ganglion cell degeneration leading to optic neuropathy (Delettre et al., 2000). In mammalian mitochondria, nuclear-encoded OPA1 ubiquitously exists in eight different isoforms, ranging from 924 to 1,014 amino acids (Olichon et al., 2007; Del Dotto et al., 2018). After import of the precursor OPA1 protein through the OMM and IMM translocases, the N-terminal mitochondrial targeting sequence (MTS) is cleaved by the mitochondrial processing peptidase (MPP), generating the membrane-anchored OPA1 long forms (L-OPA1). The L-OPA1 may be further proteolytically processed at the N terminus by two IMM peptidases, OMA1, and *i*-AAA protease YME1L, to produce the short forms of OPA1 (S-OPA1) that are soluble in the IMS (MacVicar and Langer, 2016). According to Anand et al., L-OPA1 is sufficient to promote mitochondrial fusion, whereas S-OPA1 is found to function in mitochondrial fission. Under normal metabolic control, YME1L is activated for L-OPA1 processing, whereas OMA1 is inactivated or undergoes an autocatalytic self-degradation (Baker et al., 2014). However, OMA1 is activated upon various stress insults and mitochondrial dysfunction, for example, dissipation of mitochondrial membrane potential (ΔΨm), which results in the complete degradation of L-OPA1 and an increase in S-OPA1, inducing mitochondrial fragmentation (Baker et al., 2014; Zhang et al., 2014).

Eight OPA1 isoforms with diverse roles are variably expressed in different tissues, with the highest levels observed in the retina, brain, testis, heart, and muscle. According to current research, the most affected tissues from the OPA1 mutation and depletion are the retina and brain, leading to a variety of neurodegenerative diseases, such as DOA, chronic progressive external ophthalmoplegia (CPEO), PD, and dementia (Ferré et al., 2005; Olichon et al., 2007; Zanna et al., 2008; Williams et al., 2012; Kushnareva et al., 2013; Carelli et al., 2015a; Santarelli et al., 2015; Zorzano and Claret, 2015). The OPA1 mutation caused DOA is the most prevalent inherited form of optic neuropathy, characterized by the gradual degeneration of retinal ganglion cells (RGCs) and the optic nerve. Over a hundred distinct OPA1 variants have been identified in patients with DOA so far (Ferré et al., 2005; Weisschuh et al., 2021). The mechanistic investigation discovered that the OPA1-involved dysregulated mitochondrial fusion, crista morphology, OXPHOS, and Ca^2+^ buffering are the primary sources of RGCs and optic nerve injury (Zanna et al., 2008; Kushnareva et al., 2013). OPA1 mutations can be substitutions, deletions, or insertions, which are spread throughout the coding region of the gene, with the majority of them clustered around the GTPase and GED domains. In DOA patients of European heritage, the c.2708delTTAG frameshift-inducing microdeletion in the GED domain is the most frequent OPA1 mutation (Zanna et al., 2008).

OMA1-mediated OPA1 processing often occurs during stress, and a low ratio of L-OPA1 to S-OPA1 disrupts mitochondrial dynamics, which is the cause of neuronal degeneration. Hence, OMA1 is a critical regulator of neuronal survival, particularly under stressful situations (Merkwirth et al., 2012; Korwitz et al., 2016). For example, ischemia-reperfusion (I/R) injury induces a dramatic increase in L-OPA1 cleavage, which results in L-OPA1 loss, fragmented mitochondrial morphology, and retinal neuron death. Overexpression of the OMA1 resistant cleavage OPA1 mutant (Opa1-ΔS1) prevented retinal neuronal I/R injury by restoring ATP production and mitochondrial networks, thus attenuating necroptosis and neuronal damage (Sun Y. et al., 2016). Another study also reports that stress-induced OPA1 processing by OMA1 promotes neuronal death and neuroinflammatory responses, whereas OMA1 ablation delays neurodegeneration by preventing stress-induced OPA1 processing in mitochondria (Korwitz et al., 2016).

#### SLC25A46

The 46th isoform of superfamily A of the solute carrier (SLC) family 25, termed SLC25A46, is an integral mitochondrial outer membrane protein that interacts physically with Mfn2, OPA1, and the mitochondrial contact site and cristae organizing system (MICOS) complex (Abrams et al., 2015; Li et al., 2019). SLC25A46 plays important roles in the regulation of mitochondrial dynamics, cristae architecture, as well as mitochondrial distribution (Abrams et al., 2015; Janer et al., 2016). SLC25A46 deficiency not only leads to hyper fused mitochondrial morphology but also abnormal crista architecture, whereas SLC25A46 overexpression induces fragmented mitochondria (Abrams et al., 2015; Janer et al., 2016), indicating that SLC25A46 acts as an independent pro-fission factor. Abnormal crista architecture and enlarged mitochondria with swollen cristae after depletion of SLC25A46 confirmed its role in crista morphogenesis (Wan et al., 2016). In addition, SLC25A46 interacts with components of the MICOS complex, which is critical for the maintenance of mitochondrial crista junctions (Janer et al., 2016). In SLC25A46-mutated Purkinje cells, mitochondria had an aberrant distribution and mobility (Abrams et al., 2015; Terzenidou et al., 2017). Moreover, SLC25A46 serves as a regulator for Mfn1/2 oligomerization and facilitates lipid transfer between the ER and mitochondria (Li et al., 2017, 2019).

SLC25A46 mutations have been linked to a wide spectrum of neurological diseases with recessive inheritance, including LS, optic atrophy, progressive ataxia, peripheral neuropathy, and lethal congenital pontocerebellar hypoplasia (Janer et al., 2016; Li et al., 2017, 2019). One clinical study reported that eight patients from four unrelated families encompassed autosomal dominant optic atrophy (ADOA) like optic atrophy, CMT-like axonal peripheral neuropathy, and cerebellar atrophy. The whole-exome sequencing of those patients found the recessive mutations in SLC25A46 (Abrams et al., 2015). The pathogenic mechanism investigation using SLC25A46 knockout mice discovered that animals lacking SLC25A46 have severe ataxia, which is mostly caused by Purkinje cell degeneration. Investigation of the underlying cellular basis found increased numbers of small, unmyelinated, and degenerated optic nerves as well as loss of RGCs, indicating optic atrophy. Cerebellar neurons having SLC25A46 mutation have large mitochondria with aberrant distribution and transport (Li et al., 2017).

### Mitochondrial fission

#### Drp1

Drp1 is encoded by the nuclear gene DNML1 and serves as a central molecular player in mitochondrial fission (Smirnova et al., 2001). During the mitochondrial fission process, Drp1 is recruited from a cytosolic pool onto the mitochondrial surface where ER marks the constriction sites (Pagliuso et al., 2018). Four mitochondrial outer membrane-localized adaptors, Mff, Fis1, MiD49, and MiD51, serve as receptors and independently recruit Drp1 onto the mitochondrial outer membrane. By self-assembling into a spiral superstructure, Drp1 wraps around and constricts mitochondrial tubules to promote the scission event (Jimah and Hinshaw, 2019). Because Drp1 is unable to complete the mitochondrial tubule scission, another GTPase Dnm2 is recruited to the mitochondrial fission site. By assembling into a collar-like structure around the constricting lipid “necks” of the budding membrane, Dnm2 mediates final membrane scission, creating individual mitochondria (Ferguson and De Camilli, 2012; Tilokani et al., 2018). Depletion of any of these fission-related proteins disrupts the mitochondrial dynamics with a reduction of mitochondrial fission, resulting in the elongated mitochondrial morphology (Ferguson and De Camilli, 2012; Losón et al., 2013).

Drp1 deletion leads to extensive mitochondria networks, and the mouse embryos fail to undergo developmentally regulated apoptosis during neural tube formation, which is lethal for the embryos. Additionally, brain-specific Drp1 ablation caused giant mitochondria in Purkinje cells and may be linked to cerebellum developmental abnormalities (Wakabayashi et al., 2009; Yamada et al., 2016). Besides protein-loss-caused neuronal degeneration, mutations of Drp1 are also able to impair self-assembly at the fission site and act in a dominant-negative manner (Chan, 2020). In neurological diseases, the Drp1 mutation appears to be de novo, heterozygous and dominantly acting. One clinical case reports that a single point mutation in Drp1 A395D causes elongated mitochondrial morphology, which may be associated with multi-system damage, including microcephaly, abnormal brain development, optic atrophy, and hypoplasia, resulting in neonatal lethality (Waterham et al., 2007). Another mutation in Drp1 (R403C) impairs mitochondrial fission *via* reducing Drp1 recruitment to mitochondria and Drp1 oligomerization at the fission site. Unlike the severe lethality of the Drp1 A395D mutation, the impairment of the missense mutation in Drp1 R403C is compatible with normal conditions for several years but develops refractory focal status epilepticus and subsequent rapid neurological degeneration later on (Fahrner et al., 2016).

The dynamic recruitment of Drp1 onto the OMM surface, assembly, and its GTPase activity are frequently regulated by several post-translational modifications like phosphorylation (Chang and Blackstone, 2010), ubiquitination (Wang et al., 2011), SUMOylation (Wasiak et al., 2007), and S-nitrosylation (Cho et al., 2009). Among them, phosphorylation of Drp1 at S600 or S579 by multiple kinases including PKA, Cdk1, ERK1/2, or Cdk5 has gotten the greatest interest (Taguchi et al., 2007; Valera-Alberni et al., 2021). Through phosphorylation of Drp1 at Ser579, Cdk5 promotes Aβ1-42-induced mitochondrial fission and mitophagy, whereas blocking Drp1 phosphorylation at Ser579 protects neurons from Aβ1-42-induced neuron degeneration (Han et al., 2017; Xu et al., 2021). In 1-methyl-4-phenylpyridinium ion (MPP^+^) treated cells, the overproduced nitric oxide (NO) triggers the phosphorylation of Drp1 Ser616 and leads to elevated mitochondrial fission. These events create a death-prone environment, which contributes to the loss of dopaminergic (DA) neurons and may explain the pathogenesis of PD (Zhang et al., 2016). Moreover, in human postmortem brains of HD patients, the elevated S-nitrosylation of Drp1 (SNO-Drp1) profoundly up-regulates the GTPase activity of Drp1, which leads to mitochondrial fragmentation, thus impairing bioenergetics and inducing synaptic damage and neuronal loss (Haun et al., 2013).

#### Mff

Mitochondrial fission factor, termed Mff, is a tail-anchored mitochondrial outer membrane protein (Gandre-Babbe and van der Bliek, 2008). Mff recruits cytosolic Drp1 to the mitochondrial surface and promotes mitochondrial fission by forming a complex with Drp1 (Otera et al., 2010; Dikov and Reichert, 2011). Despite the fact that four canonical receptors are involved in Drp1 recruitment, Mff depletion without disturbance of the other three receptors still results in a reduced number of Drp1-positive foci at the OMM, accompanied by impairment of mitochondrial fission, showing severely elongated and interconnected mitochondria (Losón et al., 2013). In contrast, Mff overexpression produces the opposite effects (Otera et al., 2010), demonstrating that Mff can function independently in Drp1 recruitment.

The loss of Mff in neurons reduces mitochondrial fission, which restricts the entrance of mitochondria into the axon and along the axonal shaft. Although Mff deletion has only a minimal impact on mitochondrial trafficking along the axon, presynaptic location, membrane potential, or their ability to generate ATP, it significantly boosts their Ca^2+^ buffering capacity. The size-dependent increase in mitochondrial Ca^2+^ uptake reduces presynaptic cytoplasmic Ca^2+^ levels, resulting in decreased neurotransmitter release and terminal axon branching during evoked activity (Lewis et al., 2018). In humans, a truncating mutation in Mff caused loss of function in mitochondrial fission is highlighted in those patients with early-onset Leigh-like basal ganglia disease, encephalopathy, seizures, developmental delay, and acquired microcephaly appearing in the first year of life, followed by dysphagia, spasticity, optic neuropathy, and peripheral neuropathy in subsequent years (Shamseldin et al., 2012; Koch et al., 2016; Panda et al., 2020).

## Dysregulation of Mitophagy

Mitophagy is an autophagic mechanism that specifically targets excess or damaged mitochondria and delivers them to lysosomes for degradation (Lemasters, 2005; Palikaras et al., 2015; Sun N. et al., 2016; Pickles et al., 2018; Onishi et al., 2021). Mitophagy is a set of processes that begins with the isolation of excess or damaged mitochondria and ends with the complete degradation of damaged mitochondria. In brief, the entire process can be completed in the following steps: (1) The isolation of excess or damaged mitochondria by fission in response to intracellular or external stimuli; (2) The recruitment and/or activation of mitophagy receptors or ubiquitin-autophagy adaptors on the mitochondrial surface, conferring selectivity for degradation; (3) The recognition of selected mitochondria by core autophagy-related proteins, mediating the isolation membrane/phagophore enclosing mitochondria; (4) The formation of spherical double-membrane structure “autophagosome”; (5) Transportation of autophagosomes to lysosomes for fusion; and (6) Complete degradation of selected mitochondria in lysosomes for recycling of contents (Ding and Yin, 2012; Wang Y. et al., 2019; Onishi et al., 2021).

Mitophagy-mediated elimination of impaired mitochondria plays critical roles in a variety of processes, including early embryonic development, cell differentiation, inflammation, and apoptosis (Onishi et al., 2021). Mitochondrial damage is particularly harmful to the nervous system. As a result, poor mitophagy could be detrimental to neuronal health. Defects in mitophagy have been linked to aging and the pathogenesis of age-associated neurodegenerative disorders, such as PD (Mouton-Liger et al., 2017; Tanaka, 2020; Malpartida et al., 2021), AD (Fang et al., 2019; Reddy and Oliver, 2019; Pradeepkiran and Reddy, 2020), ALS (Harding et al., 2021; Madruga et al., 2021), frontotemporal dementia (FTD; Ruffoli et al., 2015) and HD (Franco-Iborra et al., 2021; Šonský et al., 2021).

**Figure 4:**
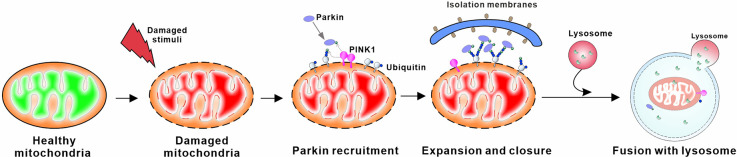
Schematic overview of PINK1/Parkin mediated mitophagy. PINK1 accumulates at the surface of damaged mitochondria when the constitutive degradation of PINK in the mitochondrial matrix is inhibited. Subsequent homodimerization of PINK1 on the OMM leads to autophosphorylation, which promotes its activation. Activated PINK1 phosphorylates ubiquitin to recruit parkin, an E3 ubiquitin ligase, to the mitochondrial membrane. PINK1 regulates the localization and activity of parkin through phosphorylation of both ubiquitin and the ubiquitin-like domain of parkin. This process leads to ubiquitination of mitochondrial proteins on the OMM that can then be bound by autophagic proteins, ultimately triggering the formation of autophagosomes that deliver the damaged mitochondria to lysosomes for degradation.

Mitophagy can be robustly induced in response to a variety of pathological stimuli (Lou et al., 2020; Pradeepkiran and Reddy, 2020). There are a number of mitophagy pathways that have been identified at present, including: (1) PTEN-induced putative kinase protein 1 (PINK1)-Parkin-mediated mitophagy; (2) Aβ and p-tau-induced mitophagy; (3) stress-induced mitophagy; (4) Parkin-independent ubiquitin-mediated mitophagy; and (5) basal mitophagy. Among all, PINK1-Parkin-mediated mitophagy is the most heavily studied and best-understood mitophagy pathway (Gautier et al., 2008; Narendra et al., 2008; Palikaras et al., 2018; Chu, 2019; Cai and Jeong, 2020). In brief, the mitochondrial imported serine-threonine protein kinase PINK1 is constitutively proteolyzed by mitochondrial proteases at the IMM under healthy conditions. Whereas, following the dissipation of mitochondrial ΔΨm, the injured mitochondria are unable to degrade PINK1, resulting in translocation of unproteolyzed PINK1 onto the OMM surface. Through phosphorylation of ubiquitin, the accumulated PINK1 recruits and activates Parkin, an E3 ubiquitin ligase (Kawajiri et al., 2010; Matsuda et al., 2010; Narendra et al., 2010; Shiba-Fukushima et al., 2012; Kane et al., 2014; Koyano et al., 2014). The activated Parkin then ubiquitinates a variety of OMM substrates, triggering the ubiquitin-proteasome system (UPS) to degrade these ubiquitinated OMM proteins. These events eventually induce the autophagy machinery, allowing the engulfment and degradation of damaged mitochondria through the above mitophagy process (Poole et al., 2010; Chan et al., 2011; Yoshii et al., 2011). In addition to mitophagy, PINK1 and Parkin regulate mitochondrial motility, which appears to be particularly important for neuronal function (Scarffe et al., 2014; Arano and Imai, 2015). These two proteins are the most well-known monogenic PD-associated genes involved in mitochondrial quality control. Therefore, loss-of-function mutations in PINK1 and Parkin are the most common known causes of autosomal recessive and early-onset PD (before the age of 45; Kitada et al., 1998; Valente et al., 2004; Thomas et al., 2007; Houlden and Singleton, 2012; Giannoccaro et al., 2017; Figure 4).

### Parkin

Parkin is an E3 ubiquitin ligase that ubiquitinates diverse substrates (Martin et al., 2011). Protein structural analysis shows that Parkin protein consists of a ubiquitin-like (Ubl) domain at the amino terminus and four RING domains: RING0, RING1, IBR, and RING2 (Shimura et al., 2000; Hristova et al., 2009; Trempe et al., 2013). This RING-in-between-RING protein structure leads to an autoinhibited state of protein activity, resulting in a very low basal activity. Mutations in protein, on the other hand, can disrupt these autoinhibitory interactions of protein domains, leading to the activation of Parkin (Trempe et al., 2013). Parkin deficiency is neurotoxic, whereas the rise of its level is neuroprotective (Hatano et al., 2009; Martin et al., 2011). More than 120 mutations in Parkin have been shown to cause autosomal recessive PD, with point mutations found in every domain of the protein (Kitada et al., 1998; Lücking et al., 2000; Mata, 2004).

The involvement of Parkin in PD was highlighted in 1997 (Matsumine et al., 1997). In a genetic linkage analysis of autosomal recessive juvenile parkinsonism (AR-JP), *PRKN*, the encoded gene of Parkin (also known as *PARK2*), was found to have a deletion at exon 4 or a large-scale deletion between exons 3 and 7 in all analyzed AR-JP patients (Matsumine et al., 1997; Kitada et al., 1998). Moreover, Parkin mutations with varied deletions or point mutations that mediate Parkin protein loss of function were soon identified in other patients of various ethnicities with early-onset PD (Hattori et al., 1998a, b; Leroy et al., 1998; Lücking et al., 1998).

### PINK1

PINK1 (also known as PARK6), the upstream protein kinase of Parkin, is one of the most diverse human protein kinases (Manning et al., 2002). PINK1 can phosphorylate both ubiquitin and the Ubl domain of Parkin on structurally protected Ser65 residue, triggering mitophagy (Kane et al., 2014; Kazlauskaite et al., 2014; Koyano et al., 2014; Wauer et al., 2015). The *PINK1* gene has eight exons that encode a protein kinase with 581 amino acids in total. It contains an N-terminal mitochondrial importing sequence and a transmembrane segment, an unconserved region, a kinase domain with three insertions in the N lobe, and a conserved C-terminal region (CTR) of unclear function and structure (Valente et al., 2004; Sim et al., 2006; Schubert et al., 2017). Under healthy conditions, the PINK1 precursor interacts with the translocase of the outer membrane (TOM) complex and is imported through the translocase of the inner membrane (TIM) complex to the IMM, where it is initially cleaved by the MPP like the importing of all traditional MTS-containing proteins (Greene et al., 2012). PINK1 is subsequently digested in its hydrophobic domain spanning the IMM by the rhomboid family protease PARL (Lazarou et al., 2012; Kato et al., 2013). The cleaved PINK1 is reversely released into the cytosol, where it is subjected to degradation by the ubiquitin ligases UBR1, UBR2, and UBR4 (Yamano and Youle, 2013). This continuous import, release, and degradation cycle yields very low to undetectable levels of PINK1 in healthy mitochondria.

However, under the damaged mitochondrial conditions, including the depolarized mitochondrial membrane potential, mutagenic or environmental stresses, OXPHOS suppression, and proteotoxicity, PINK1 import into the IMM where MPP and PARL reside is blocked, resulting in the prevention of PINK1 processing. Instead, the uncleaved PINK1 accumulates on the OMM, flagging them for elimination. Homodimerization of PINK1 on the OMM causes autophosphorylation events, which promote its kinase activation and facilitate binding to the substrates Parkin and ubiquitin (Lazarou et al., 2012; Pickrell and Youle, 2015). Due to its propensity to rapidly accumulate and activate in response to mitochondrial stress, PINK1 acts as a mitochondrial damage sensor. The mutation of the PARK6 locus on the short arm of chromosome 1 was discovered in a large Italian family with autosomal recessive early-onset parkinsonism in 2001, and the *PINK1* gene is suggested to be one of the causal genes that can result in parkinsonism (Valente et al., 2001). A number of PINK1 mutations, including point mutations, frameshift, and truncating mutations, have been further reported in patients with PD (Hatano et al., 2004; Rogaeva et al., 2004; Rohé et al., 2004).

## Dysregulation of Mitochondrial Genome Maintenance

Mitochondria possess their own circular genomic DNA (mtDNA), which encodes 13 core protein subunits of complexes I, III, IV, and V, as well as 22 transfer RNAs (mt-tRNAs) and two ribosomal RNAs (mt-rRNAs; mt-rRNAs; Clayton, 2000; Park and Larsson, 2011; Chocron et al., 2019). Due to multiple copies of mtDNA (approximately ~1,000–10,000 copies per cell), the absence of complex chromatin organization, limited mtDNA repair activities, or proximity to the ROS as a result of being close to the mitochondrial ETC, thus mutations in mtDNA occur far more frequently in mtDNA than in nDNA (~10–17-fold higher mutation rate compared to nDNA). Both deletions and point mutations can occur in the mtDNA (Marcelino and Thilly, 1999; Su et al., 2010; Tuppen et al., 2010). In addition, mtDNA mutagenesis in humans becomes more common as people get older (Bender et al., 2006). In neurons, aberrant mtDNA including primary mutations in the mtDNA itself as well as reduced mtDNA copy number contributes to bioenergetic impairments, reduced synaptic function, and an increased risk of degeneration (Keeney and Bennett, 2010).

**Figure 5:**
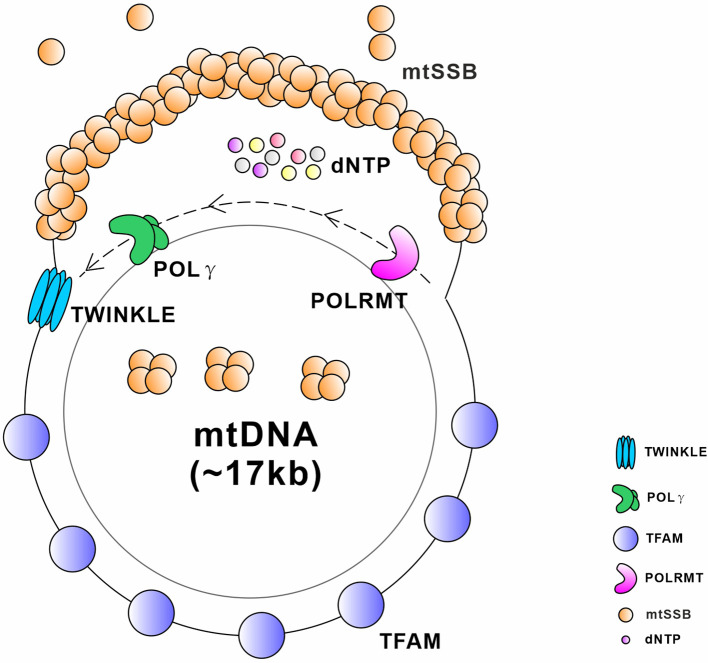
Schematic diagram of critical proteins required for the replication and transcription of mtDNA. TWINKLE is an mtDNA helicase that catalyzes the unwinding of duplex DNA using energy from NTP hydrolysis. Mitochondrial single stranded DNA-binding protein (mtSSB) exists as a stable tetramer and binds single-stranded DNA intermediates that form transiently during genome maintenance. DNA polymerase gamma (POLγ) is a DNA polymerase that synthesizes a nascent strand using one of the unwound parental template strands at the replication fork. Mitochondrial transcription factor A (TFAM) is a master regulator of mitochondrial transcription initiation and mtDNA replication that binds the promoter region of mtDNA in a sequence-specific manner and thereby initiates promoter-specific transcription or replication from the light-strand promoter. Mitochondrial RNA polymerase (POLRMT) is an RNA polymerase that executes the transcription of the mtDNA.

Unlike the nuclear-encoded genome DNA, mtDNA does not encode any genes involved in its DNA maintenance or repair. Therefore, the integrity of mtDNA is entirely dependent on the nuclear-encoded proteins (Zinovkina, 2018; Allkanjari and Baldock, 2021). Any defects in mtDNA maintenance or repair machinery, including not only the deficiencies in the related proteins themselves but also the abnormal mitochondrial importing machinery, typically result in secondary multiple deletions, duplications, or depletion of mtDNA. These changes cause subsequent poor mitochondrial respiration and dysfunction, which are linked to a broad spectrum of mitochondrial and age-related diseases (Larsson, 2010; Nunnari and Suomalainen, 2012; Figure 5 and Table 3).

**Table 3:** Neurological diseases caused by protein variants involved in mitochondrial genome maintenance.

**Protein name**	**Pathogenic mutations or changes**	**Neurological disorders**	**References**
TWINKLE	p.Ser369Pro, p.Leu381Pro p.Arg354Pro, p.Ala359Thr p.Ile367Thr, p.Val368Ile p.Arg374Gln, p.Ala475Thr	PEO	Korhonen et al. (2008), Liu et al. (2008), and Peter et al. (2019)
	p.Ala318Thr, p.Thr457Ile p.Tyr508Cys	IOSCA Epileptic encephalopathy	Hakonen et al. (2008) and Lonnqvist et al. (2009)
POLγA	p.Ala467Thr, p.Arg597Trp p.Thr748Ser, p.Arg627Trp p.His932Tyr, p.Gly1015Arg p.Arg1096Leu	AHS, MELAS, MEMSA, SANDO	Neeve et al. (2012), Rajakulendran et al. (2016), Abdoh (2019), and Parada-Garza et al. (2020)
	p.Trp748Ser p.Arg964Cys	Neurodegenerative phenotypes, like ataxia, parkinsonism, and seizures	Van Goethem et al. (2003), Luoma et al. (2004), Hakonen et al. (2005), and Hsieh et al. (2019)
p.Ser1181Asn		Neuromyopathic phenotype	Dohrn et al. (2022)
POLγB	p.Asp433Tyr	Optic atrophy, movement disorders	Dosekova et al. (2020)
TFAM	p.Arg232Cys	Seizures, intellectual disability, and hearing loss	Ullah et al. (2021)
	p.Ser12Thr	PD	Alvarez et al. (2008)
Decreased level		HD, AD	Kim et al. (2010) and Kang et al. (2018)
POLRMT	p.Pro566Ser, p.Asp870Asn p.Ser1193Phe, p.His250Asp p.Pro742_Pro747del p.Gly881_Lys883del p.Ser611Phe, p.Phe641Leu p.Cys925*, p.Pro810Ser p.Gln149*, p.Arg1013Cys p.Gln149*, p.Arg1013Cys	Global developmental delay, hypotonia, short stature, and speech/intellectual disability	Oláhová et al. (2021)

### TWINKLE

TWINKLE helicase is the main helicase in mitochondria encoded by the *TWNK* gene, serving as the only replicative helicase required for mtDNA replication (Peter and Falkenberg, 2020). Helicases are a collection of motor proteins that catalyze the unwinding of duplex DNA/RNA using energy from nucleotide triphosphate (NTP) hydrolysis. They function in almost all nucleic acid transactions, including DNA replication, transcription, translation, recombination, and DNA repair (Donmez and Patel, 2006; Singleton et al., 2007; Lohman et al., 2008; Pyle, 2008). Based on structure and sequence similarities, helicases are classified into six superfamilies (SF1–6; Singleton et al., 2007; Fairman-Williams et al., 2010), and TWINKLE is a member of the SF4 helicase superfamily, which shares five conserved helicase motifs and forms a ring-shaped higher-order structure to unwind mtDNA (Spelbrink et al., 2001). TWINKLE is hexameric and each monomer is comprised of an N-terminal domain (NTD) and a C-terminal domain (CTD), which are joined by a flexible linker helix. The five SF4 helicase motifs are located in the highly conserved CTD domain (Singleton et al., 2007). The activity of TWINKLE helicase is stimulated by interacting with mitochondrial single-stranded DNA (ssDNA)-binding protein (mtSSB; Korhonen et al., 2003; Oliveira and Kaguni, 2011). In order to efficiently initiate DNA unwinding, TWINKLE requires a fork structure comprising a single-stranded 5’ DNA end and a short 3’ tail (Patel and Picha, 2000; Korhonen et al., 2003).

TWINKLE mutations were initially detected in autosomal dominant progressive external ophthalmoplegia (PEO), a neurodegenerative disease that largely affects the muscles controlling eye movement (Spelbrink et al., 2001; Iakovenko et al., 2020). Up to 40 point mutations in the *TWNK* gene have been identified in patients with PEO, and these mutations can occur at both the primase and helicase domains as well as the linker region of the TWINKLE protein (Peter and Falkenberg, 2020). These mutations impair the helicase activity of TWINKLE, which is likely to impede mtDNA replication and lead to the gradual accumulation of large mtDNA deletions over time (Zeviani et al., 1989; Peter and Falkenberg, 2020). For example, TWINKLE mutations in the linker region (p.A359T, p.I367T, p.S369P, p.R374Q, and p.L381P) disrupt the ring-shaped structure, causing ATP hydrolysis to be reduced and DNA helicase function to be lost (Korhonen et al., 2008; Peter et al., 2019).

Apart from PEO, mutations in the *TWNK* gene have been discovered in a variety of mitochondrial illnesses, including mtDNA depletion syndromes (MDSs), Perrault syndrome, infantile-onset spinocerebellar ataxia (IOSCA), and other ataxia neuropathies (Hudson et al., 2005; Hakonen et al., 2008; Lonnqvist et al., 2009; Van Hove et al., 2009; Fratter et al., 2010). For example, several *TWNK* mutations like p.A318T, p.T457I, or p.Y508C, are supposed to disrupt the NTP binding/hydrolysis and/or oligomerization, which leads to severe mtDNA depletion and impaired OXPHOS activity, manifesting clinically as seizures, developmental delay, and peripheral neuropathy (Hakonen et al., 2008; Lonnqvist et al., 2009).

### Polymerase-γ (POLγ)

POLγ is the most important DNA polymerase executing mtDNA replication and consists of two subunits, a catalytic subunit POLγA, and a homodimeric accessory subunit POLγB (Ropp and Copeland, 1996). POLγA, which is encoded by the *POLG* gene, contains an amino-terminal exonuclease domain coupled to the carboxy-terminal polymerase domain by a linker region. The *POLG2*-encoded POLγB provides high processivity of the POLγ complex by accelerating its DNA binding affinity (Johnson et al., 2000; Young et al., 2015). B-stimulated TWINKLE helicase activity is thus required to unwind the mtDNA duplex at the replication fork, where POLγ synthesizes the nascent strand using one of the unwound parental template strands (Clayton, 1982; Bogenhagen and Clayton, 2003; Bowmaker et al., 2003).

*POLG* mutations are the most common cause of mitochondrial disease, particularly mitochondrial epilepsy, polyneuropathy, ataxia, and PEO (Rahman and Copeland, 2019). More than 300 pathogenic mutations have been mapped to the *POLG* gene based on the Human DNA Polymerase Gamma Mutation Database, and both homozygous and heterozygous POLγA mutations have been found in individuals ranging from newborns with myocerebrohepatopathy spectrum (MCHS) to the elderly with Parkinsonism (Tzoulis et al., 2013; Hikmat et al., 2017; Rahman and Copeland, 2019). Multiple mtDNA deletions and depletion are two of the most common consequences of POLG mutations (Rahman and Copeland, 2019). The same *POLG* mutation can often lead to deletions and depletion of mtDNA, or both, which may result in different diseases. For example, the homozygous mutation in POLγA p.A467T has been observed in patients with AHS (Alpers-Huttenlocher syndrome), MELAS, MEMSA (Myoclonic Epilepsy, Myopathy, and Sensory Ataxia), and SANDO (sensory ataxia neuropathy dysarthria and ophthalmoplegia), accompanied by a profound mtDNA depletion or deletions (Neeve et al., 2012; Rajakulendran et al., 2016). Therefore, it is difficult to predict the possible phenotype on the basis of *POLG* mutations. Despite the wide-ranging mitochondrial-related sickness, there are still common clinical features. For example, POLγA mutations in the spacer domain, such as p.W748S, have been found to be a significant cause of inherited neurodegenerative phenotypes, like ataxia, parkinsonism, and seizures (Van Goethem et al., 2003; Luoma et al., 2004; Hakonen et al., 2005).

In comparison to the POLγA-related neurological diseases, a relatively less neuronal pathology is linked to the mutation in POLγB. However, one research reported that one patient with childhood-onset and progressive neuro-ophthalmic manifestation with optic atrophy, mixed polyneuropathy, spinal and cerebellar ataxia, and generalized chorea, is caused by a homozygous missense mutation in POLG2 p.D433Y, which is associated with mtDNA depletion (Dosekova et al., 2020).

### Mitochondrial transcription factor A (TFAM)

TFAM is a high-mobility group-box (HMG) protein encoded by the nuclear genome, which binds to mtDNA in a sequence-specific or non-specific manner. It functions as a master regulator of mitochondrial transcription initiation and mtDNA copy number (Ekstrand, 2004; Campbell et al., 2012; Kang et al., 2018). TFAM binds to the promoter region of mtDNA in a sequence-specific manner and thereby initiates promoter-specific transcription or replication from the light-strand promoter (Gustafsson et al., 2016; Ramachandran et al., 2017). In a non-sequence-specific manner, TFAM binds non-specifically to all sequences of mtDNA, which plays a role as an mtDNA packaging factor that can bind, wrap, and bend the mitochondrial genome into nucleoid-like structures (Kaufman et al., 2007; Kukat et al., 2015).

TFAM protein levels in the CNS were shown to be reduced by ~43% in some AD, PD, and HD patients, demonstrating the association of TFAM abnormalities with a variety of neurodegenerative diseases (Sheng et al., 2012; Kang et al., 2018). For example, TFAM levels in brain lysates of HD patients present significant grade-dependent reductions, with a 15% drop in Grade 2 HD, 32% in Grade 3 HD, and 41% in Grade 4 HD. The decreased TFAM levels are accompanied by mitochondrial deletion as well as aberrant mitochondrial morphogenesis and dynamics (Kim et al., 2010). Besides the lower TFAM levels seen in patients with neurological diseases, TFAM gene mutations or polymorphisms, primarily from two TFAM mutations, rs1937, and rs2306604, are also supposed to increase the risk of PD (Gaweda-Walerych et al., 2010; Gatt et al., 2013), AD (Günther et al., 2004; Zhang et al., 2011; Lillenes et al., 2017), and HD progression (Lillenes et al., 2017).

In mice, heterozygous TFAM mutations (TFAM^+/−^) result in a reduction in mtDNA copy number, but homozygous TFAM mutations (TFAM^−/−^) are embryonically lethal (Larsson et al., 1998). Moreover, the generated CaMKII neuron-specific *Tfam* knockout mice through the Cre-loxP system show a remarkably reduction of mtDNA copy number and mtRNA levels in the neocortex at 2 and 4 months of age, respectively. The neuropathological consequences of the diminished mitochondrial genome in *Tfam* knockout mice are progressive nerve cell loss, apoptosis, gliosis in the neocortex and hippocampus, and a low induction of antioxidant defenses (Sörensen et al., 2001). Additional studies show that TFAM knockout in neurons results in reduced mtDNA expression and respiratory chain deficiency in midbrain DA neurons, which, in turn, leads to a Parkinson’s-like neurodegenerative phenotype with adult-onset of slowly progressive impairment of motor function accompanied by the formation of intraneuronal inclusions and dopamine nerve cell death (Ekstrand et al., 2007). These investigations reveal the crucial roles of TFAM in the maintenance of the mitochondrial genome, and that TFAM deletion in neurons would render neurons more sensitive to any impairments, leading to neurodegenerative phenotypes.

### Peroxisome proliferator-activated receptor-γ coactivator 1α (PGC-1α)

PGC-1α is a transcriptional coactivator and a master inducible upstream regulator of mitochondrial biogenesis functioning in both the nucleus and mitochondria (Wu et al., 1999; Aquilano et al., 2010; Safdar et al., 2011). In the nucleus, PGC-1α exerts its pleiotropic effects in mediating mitochondrial biogenesis by binding and working together with other transcription factors, such as peroxisome proliferator-activated receptors (PPARs), nuclear respiratory factors (NRF1 and NRF2), and cAMP response element-binding protein (CREB; Schreiber et al., 2004; Arany et al., 2005; Wu et al., 2006; Nirwane and Majumdar, 2018; Liu et al., 2021). For example, PGC-1α activates the transcriptional factor NRF1, which subsequently binds to the specific promoter site of TFAM and regulates its expression in the nucleus. Elevated TFAM expression eventually promotes the transcription of the mtDNA (Wu et al., 1999). Interestingly, PGC-1α was also found to reside in the mitochondria and form a multiprotein complex with sirtuin 1 and TFAM, suggesting that they may play a coordinating role in the regulation of mitochondrial biogenesis (Aquilano et al., 2010).

PGC-1α expression is closely correlated with the survival of a variety of neurons, including cholinergic, glutamatergic, dopaminergic, and GABAergic synapses in various regions of the CNS (Zhao et al., 2011; Arnold et al., 2014; Bartley et al., 2015; Jiang et al., 2016; Panes et al., 2022). And growing evidence reveals that impaired PGC-1α expression and/or function is a frequent underlying cause of mitochondrial malfunction in neurological illnesses like PD, HD, and ALS (Johri et al., 2013; Bayer et al., 2017; Yang et al., 2020; Piccinin et al., 2021). For example, PGC-1 levels are found to be lower in HD postmortem brains (Reddy et al., 2009). Investigation of a large cohort of PD patients and age-matched controls by multiplexed probe sequencing reveals that two PGC-1α variants (rs6821591 CC and rs2970848 GG) are associated with the risk of PD onset (Clark et al., 2011). Therefore, it is essential to maintain PGC-1α level in a proper range and its normal function.

### Mitochondrial RNA polymerase (POLRMT)

After TFAM binds to a position upstream of the transcription start site to initiate mtDNA transcription, TFAM recruits the POLRMT to the promoters, followed by the recruitment of another transcription factor TFB2M to fully assemble the transcription initiation complex (Shi et al., 2012; Barshad et al., 2018). POLRMT is an RNA polymerase executing transcription of the mtDNA. Additionally, during mtDNA replication, POLRMT is also required to initiate mtDNA replication by synthesizing the RNA primers (Fusté et al., 2010; Posse et al., 2019). POLRMT comprises four primary domains: the N-terminal extension (also known as the “tether helix”), a pentatricopeptide repeat (PPR) domain, the NTD, and the CTD (Ringel et al., 2011). The N-terminal extension domain is involved in the interactions between POLRMT and TFAM, which is crucial for anchoring the active site of POLRMT near the transcription start site, whereas the NTD is involved in the contact of POLRMT with TFB2M. which leads to the melting of the DNA duplex and the formation of the open initiation complex, where de novo RNA synthesis can begin (Hillen et al., 2017; Oláhová et al., 2021).

Eight patients with diverse mutations in the *POLRMT* gene at different points of sequence, including p.P566S, p.S1193F, p.D870N, p.P742_P747del, p.H250D, p.S611F, p.F641L, p.C925X, p.P810S, p.F149X, p.R1013C, p.G881_K883del, cause a broad range of neurological manifestations, such as developmental delays including fine motor, gross motor, language, social/behavioral, and thinking/intellectual. These POLRMT mutations induce a defect in mitochondrial mRNA synthesis but without mtDNA deletions or copy number abnormalities (Oláhová et al., 2021).

## Dysregulation of Mitochondrial Import Machinery

**Figure 6:**
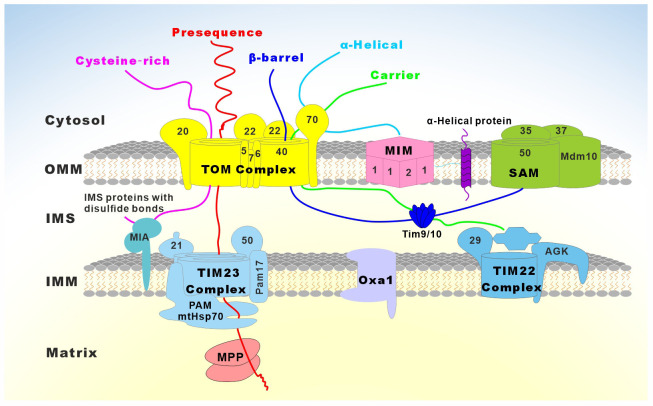
Overview of the major mitochondrial protein import pathways. Mitochondrial precursor proteins are synthesized in the cytosol, transported to the mitochondria, and directed to the correct mitochondrial compartments. Five major protein import pathways have been identified so far: (1) Presequence-carrying preproteins are imported through the translocase of the outer mitochondrial membrane (TOM) and the translocase of the inner mitochondrial membrane (TIM23) complexes. Proteins containing a hydrophobic sorting signal are released into the inner mitochondrial membrane (IMM), while hydrophilic proteins are imported into the matrix with the help of the presequence translocase-associated motor (PAM) complex. Membrane potential across the IMM is essential for the entry of presequences into the matrix. The presequences are cleaved by the mitochondrial processing peptidase (MPP) and additional proteolytic processing occurs by intermediate cleaving peptidases. Presequence-carrying precursors that are integrated into the IMM are either directly released from the TIM23 complex or transported into the matrix, followed by further insertion into the IMM with the help of Oxa1; (2) Cysteine-rich proteins of the intermembrane space (IMS) are imported by TOM and the mitochondrial IMS import and assembly (MIA) system, which inserts disulfide bonds in the imported proteins; (3) The precursors of β-barrel proteins are translocated through the TOM complex to the TIM9/10 in the IMS and are inserted into the outer mitochondrial membrane (OMM) by the sorting and assembly machinery (SAM); (4) The precursors of metabolite carriers of the IMM are imported *via* the TOM complex, small TIM9/10 chaperones, and the carrier translocase TIM22 complex; and (5) Some proteins with α-helical transmembrane segments are inserted into the OMM by the mitochondrial import (MIM) complex.

In mammalian mitochondria, 99% of proteins are encoded by nuclear genes and synthesized on cytosolic ribosomes as precursor proteins, most of which are subsequently recognized and imported into mitochondria through a common entry gate, the translocase of TOM complex, and then sorted to the destined subcompartments of mitochondria *via* five major protein import pathways (Figure 6; Schmidt et al., 2010; Wiedemann and Pfanner, 2017): (1) The classical import pathway is termed the “presequence pathway”, which is characterized by cleavable N-terminal mitochondrial targeting sequences (MTSs). The vast majority of matrix proteins and many inner membrane-localized proteins are dependent on the presequence pathway for mitochondrial entry (Abe et al., 2000; Vögtle et al., 2009). After passing through the TOM complex, those presequence-carrying precursors are subsequently imported by presequence translocase of the inner membrane TIM23 (Mokranjac and Neupert, 2015). The presequence translocase-associated motor (PAM) drives protein translocation into the matrix, where the MTSs are proteolytically removed by an MPP (Hawlitschek et al., 1988). In contrast to the presequence pathway, the precursor proteins of the other four import pathways do not carry cleavable MTS, but instead possess different kinds of internal MTSs (Schmidt et al., 2010; Wiedemann and Pfanner, 2017); (2) SAM pathway: the precursors of β-barrel proteins are translocated through TOM and bind the small TIM chaperones TIM9/10 in the intermembrane space, where the sorting and assembly machinery (SAM) inserts them into the outer membrane (Paschen et al., 2003; Wiedemann et al., 2003); (3) The mitochondrial IMS import and assembly (MIA) pathway: the cysteine-rich precursors are kept in a reduced state in the cytosol, imported by the TOM complex, and oxidized by the MIA system (Chacinska et al., 2004); (4) Carrier pathway: precursors of the multispanning hydrophobic carrier proteins of the inner membrane are imported *via* TOM, small TIM chaperones TIM9/10, and the carrier translocase TIM22 (Sirrenberg et al., 1996, 1998); and (5) The mitochondrial import (MIM) pathway: a number of proteins with α-helical transmembrane segments are inserted into the outer membrane by the MIM complex (Becker et al., 2008; Popov-Čeleketić et al., 2008).

Mitochondrial importing machinery is essential for protein translocation and is deeply integrated into a functional network of mitochondrial bioenergetics, dynamics, and morphology, as well as protein quality control and interaction with other organelles. Thus, mitochondrial import defects can potentially induce all types of mitochondrial dysfunction. As a result, an increasing number of cases of defects in proteins involved in mitochondrial import have been associated with a spectrum of disorders, including different kinds of neuropathies (Nicolas et al., 2019; Palmer et al., 2021; Figure 6 and Table 4).

**Table 4:** Neurological diseases caused by protein variants involved in mitochondrial import machinery.

**Protein name**	**Pathogenic mutations or changes**	**Neurological disorders**	**References**
Tom70	p.Thr607Ile p.Ile554Phe	Hypotonia, hyperreflexia, ataxia, dystonia, and white matter abnormalities	Dutta et al. (2020)
	p.Thr265Met, p.Ala582Val	Developmental delay	Wei et al. (2020b)
Tim50	p.Arg217Trp, p.Thr252Met	Intellectual disability and seizure disorder	Shahrour et al. (2017)
	p.Ser112X p.Gly190Ala	Encephalopathy	Reyes et al. (2018)
Tim22	p.Val33Leu, p.Tyr25X	Neuromuscular presentation and persistently elevated CSF	Pacheu-Grau et al. (2018)
Tim8a	p.Met1Leu, p.Met39Argfs26 p.Lys50Glnfs12, p.Gln18X p.Cys66Trp, p.Glu24X p.Val25 × , p.Arg80X p.Met1Ile, p.Cys43Valfs22 p.Gln38 × , p.Leu78Serfs21 p.Glu45del, p.Gln28X	DDON	Tranebjærg et al. (1995), Tranebjærg et al. (2001), Jin et al. (1996), Ujike et al. (2001), Wang et al. (2019), Neighbors et al. (2020), and Song et al. (2021)
Oxa1	p.Ser170Glnfs*18 p.Cys207Phe	Encephalopathy, hypotonia and developmental delay	Thompson et al. (2018)
MPP	p.Arg175Cys, p.Ala201Pro p.Arg175His, p.Val177Gly p.Ile422Thr	Lack of speech Neurodegeneration in Early Childhood	Vögtle et al. (2018)
	p.Ala377Thr, p.Ser96Leu p.Gly515Arg	Cerebellar ataxias	Jobling et al. (2015)
	p.Gly356Ser, p.Ala377Thr	Encephalopathy	Joshi et al. (2016)

### Tom70

Tom70 is an adaptor protein of the TOM complex that loosely attaches to the TOM complex and plays a major role in the import of non-cleavable hydrophobic precursor proteins such as the carrier precursors (Brix et al., 1999; Young et al., 2003). Tom70 binds the precursors-chaperone complex, facilitating the transfer of precursors to the channel-forming protein Tom40, where the precursors are translocated across the outer membrane (Hill et al., 1998; Wiedemann and Pfanner, 2017). Two children with distinct point mutations in Tom70, p.T607I, and p.I554F, present common features including significant white matter abnormalities, hypotonia, hyperreflexia, dystonia, and cognitive deficits. Causative investigation of Tom70-induced neurological abnormalities in Drosophila reveals that homozygous Tom70 null flies are pupal lethal. Tom70 knockdown impairs synaptic transmission and results in degenerative eye phenotype, indicating that Tom70 is required in neuronal maintenance (Dutta et al., 2020). Another study on a patient with Tom70 heterozygous mutations [(p.T265M) and (p.A582V)] found OXPHOS complex impairments, particularly complex IV (Wei et al., 2020b).

### Tim50

Tim50 is a component of the Tim23 translocase complex in the IMM. Tim50 exposes its domains to the IMS and functions as a presequence receptor that binds preproteins emerging on the intermembrane space side of Tom40-Tom22 (Lytovchenko et al., 2013; Rahman et al., 2014). By cooperating with the regulatory subunit Tim21, Tim50 interacts with the N-terminal intermembrane space domain of Tim23, the channel-forming protein of the TIM23 complex, and regulates the open or closed state of the Tim23 channel. In the absence of preproteins, Tim50 helps keep the Tim23 channel in a closed state. Upon binding of a presequence, the Tim23 channel is activated and opened (Truscott et al., 2001; Meinecke et al., 2006). Using whole-exome sequencing, researchers discovered that two homozygous missense mutations in the *TIMM50* gene, p.R217W, and p.T252M, cause severe intellectual disability and seizure disorder in four patients (Shahrour et al., 2017). Moreover, a compound heterozygous mutation in *TIMM50* (p.S112X and p.G190A) in an infant patient causes rapidly progressive and severe encephalopathy. These mutations induce the reduction of the Tim50 level, accompanied by lower steady-state levels of several components of OXPHOS and enhanced ROS production (Reyes et al., 2018).

### Tim22

Tim22 is a channel-forming protein of the TIM22 complex, mediating the insertion of precursor proteins into the inner membrane in a Δψ-driven manner (Wiedemann and Pfanner, 2017). Tim22 with the p.V33L mutation disrupts the formation of the translocase complex and compromises levels of metabolite carrier proteins within the inner membrane, which may cause an imbalance of mitochondrial metabolites and lead to a secondary dysfunction of OXPHOS. A case of compound heterozygous variants in Tim22 (a point mutation variant p.V33L and a premature truncated variant p.Y25X) in a young patient presents delayed myelination, neuromuscular illness with hypotonia, gastroesophageal reflux disease, consistently elevated lactate in the serum, and cerebrospinal fluid (CSF; Pacheu-Grau et al., 2018).

### Tim8a

Tim8 is a member of the small Tim chaperones that dynamically interacts with the TIM22 complex to transfer the precursor proteins for sorting and insertion into the inner membrane by preventing protein aggregation within the aqueous IMS (Bauer et al., 1999). In humans, there are two Tim8 isoforms, Tim8a and Tim8b, which have been discovered to play a role in the assembly of complex IV (Gentle et al., 2007; Kang et al., 2019). Compared to Tim8b, Tim8a is more strongly expressed in the human brain (Kang et al., 2019). In accordance with its distribution, diverse mutations in the *TIMM8A* gene (p.M1L, p.E24X p.Q28X, p.Q38X, p.M39fs, p.C43fs, p.K50fs, p.C66W, p.R80X) cause Mohr-Tranebjaerg syndrome (also called deafness-dystonia-optic neuronopathy [DDON]), an X-linked recessive neurodegenerative disorder characterized by progressive sensorineural hearing loss, dystonia, cortical blindness, and dysphagia (Tranebjærg et al., 1995, 2000; Jin et al., 1996; Ujike et al., 2001; Neighbors et al., 2020).

### Oxa1

Oxa1 is a member of the YidC/Alb3/Oxa1 insertase family and is implicated in protein insertion machinery by exporting proteins from the matrix into the inner membrane (Hennon et al., 2015). A large number of inner membrane-localized proteins, such as the TIM22 complex, CI, CIV, and F_1_F_o_-ATP Synthase, are dependent on the Oxa1 involved import machinery (Bonnefoy et al., 1994; Altamura et al., 1996; Stiburek et al., 2007). Due to the crucial role of Oxa1 in the assembly of OXPHOS complexes, biallelic variants in OXA1L (p.S170Qfs*18 and p.C207F) in a patient caused a profoundly decreased level of OXPHOS complexes I, IV, and V, which leads to severe illness with hypotonia, severe encephalopathy, and developmental delay in early childhood (Thompson et al., 2018).

### MPP

MPP is a heterodimeric peptidase that is comprised of MPPα and MPPβ residing in the matrix (Hawlitschek et al., 1988; Taylor et al., 2001; Gakh et al., 2002). MPP proteolytically cleaves off the N-terminal presequences of most precursor proteins that are fully translocated to the matrix as well as precursors in transit to the inner membrane or the intermembrane space (Vögtle et al., 2009; Wiedemann and Pfanner, 2017). The larger MPP subunit MPPα is likely to be engaged in substrate recognition, whereas the smaller MPP subunit MPPβ is the catalytic subunit that removes the MTS of freshly imported precursor proteins (Braun et al., 1992; Greene et al., 2012). In humans, mutations in the MPPα encoding gene *PMPCA*, including the homozygous missense mutation (p.A377T) and the compound heterozygous mutations (p.S96L and p.G515R), have been uncovered in patients with non-progressive and slowly progressive cerebellar ataxias. Moreover, all patients showed impaired processing of frataxin, a well-known substrate of MPP, highlighting the tight association of MPP with ataxia-related illness (Choquet et al., 2016). Besides MPPα, biallelic mutations in the encoding gene of MPPβ, *PMPCB* (p.R175C/p.A201P, p.V177G/p.R175H, p.I422T, p.E396D), caused a complex and severe neurological phenotype of neurodegeneration and cerebellar atrophy in early childhood (Vögtle et al., 2018). Both *PMPCA* and *PMPCB* gene mutations result in a strong increase in the intermediate form of frataxin, a conserved mitochondrial protein that is implicated in the Fe-S cluster assembly in mitochondria. The reduction of mature frataxin protein impairs the biogenesis of Fe-S clusters, such as Fe-S cluster-containing OXPHOS complexes (Stemmler et al., 2010; Choquet et al., 2016; Vögtle et al., 2018).

## Dysregulation of Mitochondrial Ion Channels

Mitochondrial ion channels are a group of integral membrane proteins mediating ionic fluxes across the mitochondrial membranes, which are driven by electrochemical gradients (Δμ_ion_; O’Rourke, 2007), such as the voltage-dependent anion channel of the outer membrane (VDAC) in the OMM and the mitochondrial calcium uniporter (MCU) in the IMM. Despite the opening of mitochondrial ion channels may be transient and tightly regulated, these ion channels are critical for diverse mitochondrial functions and cell survival (O’Rourke, 2007; Szabo and Zoratti, 2014; Urbani et al., 2021). Growing evidence reveals that malfunction of mitochondrial ion channels are linked to aging and diseases like PD and AD (Liao et al., 2017; Surmeier et al., 2017; Shoshan-Barmatz et al., 2018; Ashrafuzzaman, 2022).

**Figure 7:**
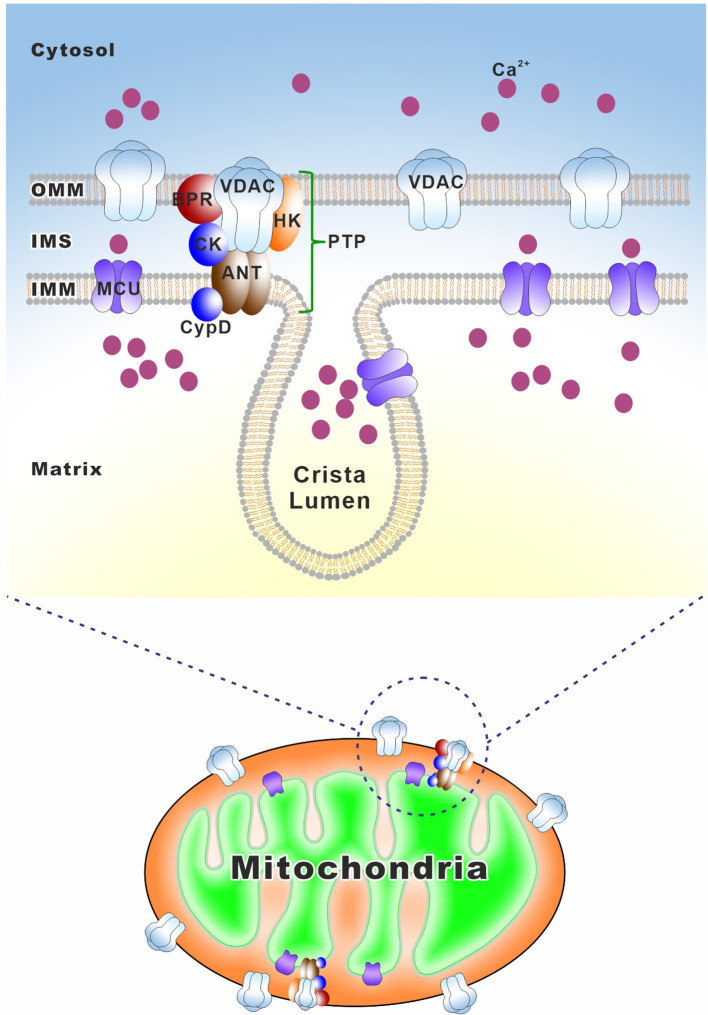
Schematic overview of mitochondrial ion channels that are implicated in neurodegeneration. Mitochondrial ion channels are a group of integral membrane proteins mediating ionic fluxes across the mitochondrial membranes. VDAC is a voltage-dependent anion channel residing in the OMM and MCU is a mitochondrial calcium uniporter in the IMM. PTP is a mitochondrial permeability transition pore that allows mitochondria to undergo a sudden increase of permeability, including VDAC in the OMM and the adenine nucleotide translocase (ANT) in the IMM. Cyclophilin D (CypD) in the matrix is a sensitizer of PTP. Peripheral benzodiazepine receptor (BPR), hexokinase (HK), and creatine kinase (CK) are the other components of PTP.

In normal conditions, the IMM is quite impermeable to ions in order to maintain the efficiency of the OXPHOS (Javadov and Karmazyn, 2007). Accumulation of ROS, imbalance in Ca^2+^ concentration, depolarization of mitochondrial membrane potential, or mutation in the mtDNA may cause a sudden increase in the IMM permeability to ions and other solutes up to 1,500 Da non-selectively. This permeability transition (PT) is caused by the opening of the permeability transition pores (PTP; Halestrap et al., 1998; Rasola and Bernardi, 2011). PTPs are large-conductance (~1 nS) pores formed by multi-proteins, such as the mitochondrial adenine nucleotide translocase (ANT) in the IMM and the VDAC in the OMM. Non-specific entry of water, ions, and solutes into mitochondria due to the opening of PTP leads to mitochondrial swelling. Although slight increases in matrix volume within the physiological range can stimulate mitochondrial function and metabolism, excessive mitochondrial swelling caused by persistent PTP opening initiates an irreversible cascade of events, including the depolarized inner membrane potential, the uncoupled OXPHOS, excess ROS production, crista unfolding, the release of stored Ca^2+^ and other apoptogenic proteins (such as Cyt *c*) into the cytosol. These events ultimately result in neuronal death (Javadov et al., 2018). In response to oxidative stress, the PTP opening also plays a causal role in the disassembling of the ETC supercomplexes (Jang et al., 2017). In PINK1-deficient midbrain neurons, the severely inhibited Ca^2+^ extrusion mediates Ca^2+^ overload inside the mitochondria and excessive ROS generation, which causes PTP opening and eventually midbrain neuronal death (Gandhi et al., 2009; Schapira, 2013). This neuronal loss is thought to play a central role in the neurodegeneration of PD (Ludtmann and Abramov, 2018; Figure 7).

### VDAC

VDAC is a highly conserved pore-forming protein located in the OMM. It is a large-conductance anion channel, providing the major pathway for transmembrane fluxes of ions and metabolites across the OMM (Shoshan-Barmatz et al., 2010; Rosencrans et al., 2021). VDAC is the most abundant OMM protein accounting for ~50% of the overall protein content and thus is responsible for 90% of the OMM permeability (Gonçalves et al., 2007; Morgenstern et al., 2017). In addition, VDAC associates with over 100 other proteins, such as proteins involved in Ca^2+^ homeostasis and apoptosis (Shoshan-Barmatz et al., 2017). Three VDAC isoforms (VDAC1, VDAC2, and VDAC3) are found in mammalian cells, and VDAC1 is the most widely expressed, followed by VDAC2 and VDAC3 (Cesar and Wilson, 2004; Messina et al., 2012). VDAC1 is linked to the toxicity of pathogenic proteins in neurodegenerative disorders, including phosphorylated tau, Aβ, α-synuclein, and γ-secretase (Magri and Messina, 2017). Furthermore, significant changes in the VDAC1 level were found in neurodegenerative illnesses such as AD, PD, ALS, and HD (Risiglione et al., 2021). For example, an aberrant VDAC1 level was found in patients with AD or Down’s syndrome (César Rosa and de Cerqueira César, 2016).

### MCU

MCU is a calcium-selective ion channel in the IMM and is responsible for low-affinity Ca^2+^ uptake into the mitochondrial matrix (Baughman et al., 2011). The MCU complex is composed of several components, including pore-forming MCU protein, MICU1 (calcium uptake protein 1, mitochondria), MICU2/3 (calcium uptake protein 2/3, mitochondria), EMRE (essential MCU regulator, mitochondria), MCUR1 (mitochondrial calcium uniporter regulator 1), and MCUb (calcium uniporter regulatory subunit MCUb, mitochondria). MCU functions as a critical pore-forming and calcium-conducting subunit, and the rest components work as regulatory subunits (Reed and Bygrave, 1974; Liao et al., 2017). MICU1 and MICU2/3 sense matrix calcium *via* their conserved calcium-binding EF-hand domains (Perocchi et al., 2010).

The mitochondrial calcium level regulates the mitochondrial activity and cellular ATP supply. Impaired Ca^2+^ homeostasis leads to mitochondrial malfunction, which promotes neurodegeneration (Kawahara and Kuroda, 2000; Wang et al., 2013; Ryan et al., 2020). For example, Aβ peptide treatment elevated the mitochondrial calcium level in cortical neurons, resulting in neurodegeneration. Inhibition of MCU activity with a specific MCU inhibitor can stop this process, indicating that the toxicity of Aβ may be partly due to its capacity to interfere with the homeostasis of mitochondrial calcium (Hedskog et al., 2013). In a multiplex consanguineous family, a homozygous truncating mutation in *MICU2* (c.42G4A, p.W14*) was found to fully segregate with a neurodevelopmental disorder characterized by severe cognitive impairment, spasticity, and white matter involvement. Patient-derived MICU2-deficient cells present impaired mitochondrial Ca^2+^ homeostasis with abnormal regulation of inner mitochondrial membrane potential as well as an increase in the vulnerability of mitochondria to oxidative stress (Shamseldin et al., 2017).

## Conclusions

A growing number of neurological illnesses are found to be linked with mitochondrial protein dysfunction. In recent years, many novel mitochondrial proteins and mutations have been identified and characterized, providing a better understanding of both mitochondrial function and dysfunction. In this review, we summarized the cellular roles of mitochondrial proteins involved in mitochondrial bioenergetics, dynamics, mitophagy, genome maintenance, importing machinery, and ion channels, as well as how aberrant mitochondrial proteins contribute to the pathogenesis of neurological disorders.

In addition to the mitochondrial protein deficits, mounting evidence reveals that numerous abnormal non-mitochondrial proteins also target mitochondria, causing mitochondrial neurotoxicity and, consequently, the onset of neurological diseases. Therefore, modulating mitochondrial function, especially regulating the mitochondrial proteome, could be a promising therapeutic avenue in treating various neurological disorders. However, many challenges remain. Although we are increasingly able to establish the genetic diagnosis in patients with mitochondrial diseases, there are still patients for whom the diagnosis is difficult. Furthermore, complications of mitochondrial disease and clinical phenotype variations in patients with similar levels of heteroplasmy are common. Thus, identifying the cause of this variation is critical for designing potentially preventative treatment strategies.

## Author Contributions

LW and ZY prepared the original draft, the figure and tables. XH, SP, and CY participated in the preparation of the manuscript. HZ supervised and revised the manuscript. QW, ZZ, and XC reviewed the manuscript. All authors contributed to the article and approved the submitted version.

## Funding

This work was supported by grants from the Academy of Finland (HZ; decision No. 323670), Jane and Aatos Erkko Foundation (HZ), and Guangxi distinguished expert funding (HZ). LW is supported by the National Natural Science Foundation of China (No. 32000719), the fellowship of China Postdoctoral Science Foundation (No. 2021M702362), and the “Post-Doctor Research project, West China Hospital, Sichuan University” (No. 2020HXBH010).
